# An immunophenotype-coupled transcriptomic atlas of human hematopoietic progenitors

**DOI:** 10.1038/s41590-024-01782-4

**Published:** 2024-03-21

**Authors:** Xuan Zhang, Baobao Song, Maximillian J. Carlino, Guangyuan Li, Kyle Ferchen, Mi Chen, Evrett N. Thompson, Bailee N. Kain, Dan Schnell, Kairavee Thakkar, Michal Kouril, Kang Jin, Stuart B. Hay, Sidharth Sen, David Bernardicius, Siyuan Ma, Sierra N. Bennett, Josh Croteau, Ornella Salvatori, Melvin H. Lye, Austin E. Gillen, Craig T. Jordan, Harinder Singh, Diane S. Krause, Nathan Salomonis, H. Leighton Grimes

**Affiliations:** 1 Division of Immunobiology, Cincinnati Children’s Hospital Medical Center, Cincinnati, OH USA; 2 Immunology Graduate Program, University of Cincinnati, Cincinnati, OH USA; 3 Yale Stem Cell Center, Yale School of Medicine, New Haven, CT USA; 4 Department of Laboratory Medicine, Yale University, New Haven, CT USA; 5 Division of Biomedical Informatics, Cincinnati Children’s Hospital Medical Center, Cincinnati, OH USA; 6 Department of Cell Biology, Yale School of Medicine, New Haven, CT USA; 7 BioLegend, Inc., San Diego, CA USA; 8 Curiox Biosystems, Inc., Woburn, MA USA; 9 Division of Hematology, University of Colorado School of Medicine, Aurora, CO USA; 10 Rocky Mountain Regional VA Medical Center, Aurora, CO USA; 11 Departments of Immunology and Computational and Systems Biology, Center for Systems Immunology, University of Pittsburgh, Pittsburgh, PA USA; 12 Department of Pediatrics, University of Cincinnati, Cincinnati, OH USA; 13 Division of Experimental Hematology and Cancer Biology, Cincinnati Children’s Hospital Medical Center, Cincinnati, OH USA

**Keywords:** Haematopoietic stem cells, Gene expression analysis, Myelopoiesis, Leukaemia

## Abstract

Analysis of the human hematopoietic progenitor compartment is being transformed by single-cell multimodal approaches. Cellular indexing of transcriptomes and epitopes by sequencing (CITE-seq) enables coupled surface protein and transcriptome profiling, thereby revealing genomic programs underlying progenitor states. To perform CITE-seq systematically on primary human bone marrow cells, we used titrations with 266 CITE-seq antibodies (antibody-derived tags) and machine learning to optimize a panel of 132 antibodies. Multimodal analysis resolved >80 stem, progenitor, immune, stromal and transitional cells defined by distinctive surface markers and transcriptomes. This dataset enables flow cytometry solutions for in silico-predicted cell states and identifies dozens of cell surface markers consistently detected across donors spanning race and sex. Finally, aligning annotations from this atlas, we nominate normal marrow equivalents for acute myeloid leukemia stem cell populations that differ in clinical response. This atlas serves as an advanced digital resource for hematopoietic progenitor analyses in human health and disease.

## Abstract (web summary)

In this Resource article, the authors integrate genomic, bioinformatic and flow cytometric data from human bone marrow to provide an atlas of hematopoietic progenitor cell states in health and disease.

## Main

Although many studies have delineated and characterized human hematopoietic stem and progenitor cells (HSPCs), a high-resolution map that integrates cellular immunophenotypes and underlying transcriptional states is lacking. HSPCs have been traditionally characterized by the combinatorial expression of surface proteins and functionally associated with their developmental potencies using colony-forming and adoptive transfer approaches. Advances in single-cell technologies have provided unprecedented insights into the diverse and complex genomic states of HSPCs and their developmental fate dynamics^1–13^. Such analyses have generated differing models of hematopoiesis, proposing discrete versus a continuum of developmental states^14^. High-resolution coupled analyses of immunophenotypic and transcriptional states of HSPCs are needed to characterize, isolate and functionally analyze stem, progenitor and transitional cell states^15^, thereby facilitating testing of competing developmental models.

Multiomics techniques allow immunophenotyping in conjunction with RNA and DNA sequencing, such as cellular indexing of transcriptomes and epitopes by sequencing (CITE-seq)^16^. CITE-seq uses antibodies conjugated to DNA oligonucleotide tags, known as antibody-derived tags (ADTs), that are captured with mRNA during droplet-based single-cell RNA sequencing (scRNA-seq)^16^. Because a theoretically infinite number of ADTs can be multiplexed in CITE-seq, this multiomics approach has the potential to explore novel markers for known transcriptionally delineated cell states as well as define distinct cellular populations with new marker combinations. Successful application of CITE-seq is critically dependent on determining relevant antibody combinations and their optimal concentrations. Although CITE-seq is being increasingly used to study normal and malignant hematopoietic progenitors, ADT panel concentrations are largely based on their applications in characterizing peripheral blood mononuclear cells (PBMCs).

To address this technical challenge and to discover, isolate and characterize rare bone marrow hematopoietic progenitors, we performed CITE-seq with titrations of 266 BioLegend TotalSeq-A antibodies on healthy adult bone marrow cells and generated data from over 300,000 single cells. Donors spanned race and sex to approximate human diversity. From this dataset, we defined a panel of 132 antibodies along with their optimal concentrations for analysis of bone marrow HSPCs. These antibodies were incorporated into Infinity Flow^17,18^, an experimental and computational workflow that exploits machine learning to impute hundreds of cell surface proteins on millions of cells. Comparison of co-normalized Infinity Flow and CITE-seq facilitated isolation of transitional cell states. Overall, our experimental framework enabled the identification of over 80 molecularly distinct early HSPC subsets, mature immune populations and stromal subsets across donors and technologies. Finally, we aligned and annotated the healthy counterparts of leukemia stem cells (LSCs) within our atlas to nominate their cellular origins and potential isolation strategies.

## Results

### Expanded repertoire of hematopoietic progenitor cell states

**Fig. 1:**
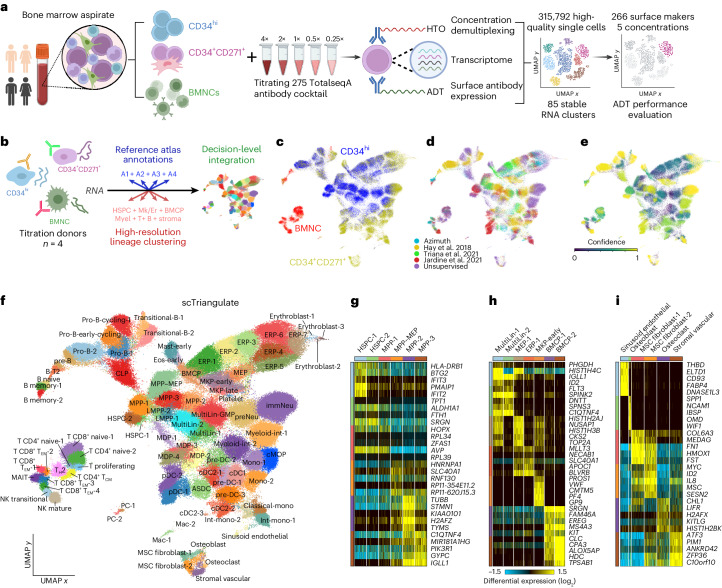
An integrative transcriptomic atlas of human bone marrow cell states. **a**, Bone marrow aspirate cell isolation and titration experimental workflow. **b**, Scheme for the integration of prior reference cell annotation labels (cellHarmony and Azimuth) with unsupervised clustering of 315,792 bone marrow cells using scTriangulate based on RNA. **c**, Uniform manifold approximation and projection (UMAP) of the source cell isolation enrichment approach for cells from four healthy donors. **d**, Final scTriangulate clusters annotated by the reference study annotation source. **e**, scTriangulate stability confidence score from the integration (Shapley confidence score). **f**, Transcriptome-defined cluster annotations based on the source clusters and marker genes. **g**–**i**, Top RNA-defined marker genes in HSPCs (**g**), presumptive multilineage progenitors (**h**) and stromal cells (**i**). Abbreviations: BMCP, basophil/mast cell progenitor; Mk, megakarocyte; Er, erythroid; Myel, myeloid; MultiLin, multi-lineage progenitor; T_EM_, effector memory T cell; T_CM_, central memory T cell; MAIT, mucosal-associated invariant T cell; MPP, multipotent progenitors; Eos, eosinophils; MKP, megakaryocyte progenitor; ASDC, AXL^+^SIGLEC6^+^ DC; Mono, monocyte; cMOP, common monocyte progenitor; Mac, macrophage; MSC, mesenchymal stem cell; Neu, neutrophil; PC, plasma cell; int, intermediate.

**Extended Data Fig. 1:**
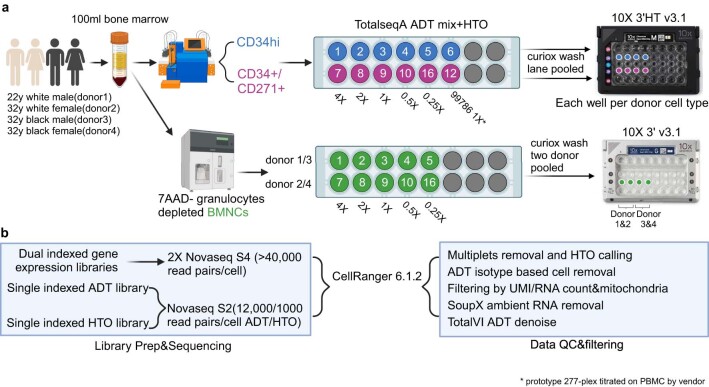
Experimental and bioinformatic of molecular titration workflow. **a**, Schematic showing sample processing, HTO multiplex strategy and 10X Chromium X loading workflow. **b**, Sequencing specifications and data pre-processing workflow. Sequencing metrics in Supplementary Table 3.

**Extended Data Fig. 2:**
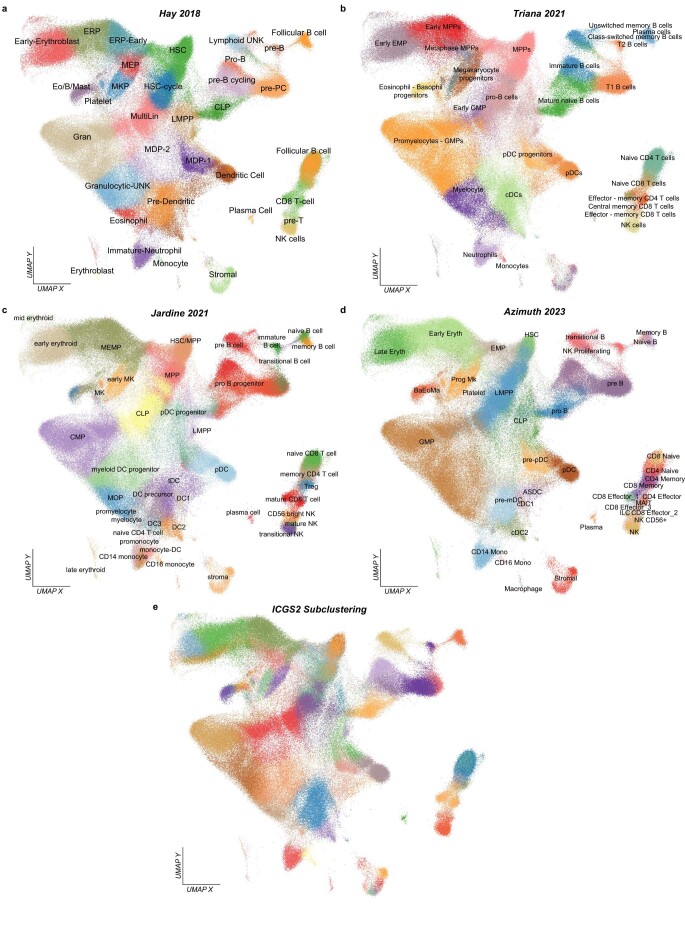
Divergent bone marrow single-cell annotations by study. **a**-**e**) UMAP of single-cell annotations by studies derived through reference label projection (cellHarmony) (**a**-**d**) or unsupervised sub-clustering (ICGS2) (**e**). The reference source annotations are indicated by first author and year of publication, where provided.

Existing single-cell bone marrow cell atlases are challenged by a lack of enriched progenitor populations, donor ethnic diversity and precision antibodies for characterization. To construct a precision panel of titrated ADTs for HSPCs, we obtained fresh bone marrow aspirates from four donors of African and European ancestry (Supplementary Table 1). We conducted CITE-seq analysis on the following populations: (1) live bone marrow nucleated cells (BMNCs), (2) CD34^+^ cells (double column; CD34^hi^) enriched by immune-magnetic sorting (magnetic-activated cell sorting) and (3) flow-through (including CD34^med/low^ cells) labeled with anti-CD271 and a single-column selection enriched for both CD271^+^ stroma and remaining CD34^med/low^-expressing cells (CD34^+^CD271^+^; Fig. 1a). As a reference set of ADTs, we used a prototype 277-plex TotalSeq-A antibody cocktail (titrated on PBMCs by the vendor). Given that many antibodies did not perform as anticipated, we worked with BioLegend to generate a customized 275-plex TotalSeq-A cocktail (comprised of 266 targeting antibodies and 9 clone-matching isotypes). The working concentrations for these antibodies were determined by the ADT signal from antibodies in the prototype PBMC-titrated mix, augmented by cytometry-based titration on CD34^+^ cells and BMNCs. The new titration panel was generated at eightfold working concentration (Supplementary Table 2) and was serially diluted to construct five concentrations (0.25×, 0.5×, 1×, 2× and 4×). We also included the prototype PBMC-titrated mix (at 1×). We used simultaneous labeling with TotalSeq-A Hashtag antibodies to denote concentration, donor and population (Fig. 1a and Extended Data Fig. 1), yielding over 315,000 single-cell profiles in which each cell could be confidently assigned to a specific concentration/donor (Supplementary Table 3 and Methods). To initially assign cellular identities in this large dataset by transcriptome, we performed label transfer (cellHarmony^19^) from four separate recently proposed bone marrow cell atlases (Extended Data Fig. 2a–d)^3,20–23^. Among the four references, we found notable discordance among cell annotations and cluster definitions. For example, stromal cells, plasma cells, eosinophil/mast cells, erythroblasts, dendritic cells (DCs) and distinct T cell/natural killer (NK) cell subsets were inconsistently annotated in these reference datasets, with some annotations supported only by a single atlas. Conversely, high-resolution unsupervised clustering (Iterative Clustering and Guide-Gene Selection version 2 (ICGS2)) predicted diverse novel cell states (Extended Data Fig. 2e). To overcome these inherent and systemic biases, we applied our recently developed integrative game theory-driven workflow called scTriangulate^24^ to assess the relative importance (stability metrics) and contribution of previously defined cell populations in bone marrow atlases in combination with our unsupervised clustering results (Fig. 1b). From this collection of 215 overlapping cluster definitions, we resolved 85 final clusters from scTriangulate with complementary evidence of transcriptional and reclassification stability (Fig. 1c–e). These resulting clusters were composed of a balanced mix of cell populations from the five different supervised and unsupervised sources. As expected, the CD34^hi^ populations were enriched for primitive HSPCs, the CD34^+^CD271^+^ populations contained mature hematopoietic progenitors and stromal cells, and the BMNC populations contained mature immune cells (Fig. 1c). Some annotations of HSPCs were based on existing literature, whereas others represented new subclusters from ICGS^25^, such as common lymphoid progenitors (CLPs) and erythroid progenitors (ERPs; Fig. 1d). These clusters exhibited different levels of Shapley-associated confidence, an indication of cluster stability, with high confidence observed among lymphoid lineages, whereas early ERPs and most stem and multilineage progenitors were stable but were called with lower confidence (Fig. 1e). In sum, we labeled most populations according to annotations from the four source cell atlases while resolving new populations based on marker genes and relative position within the low-dimensional embeddings (Fig. 1f). Through this analysis, we confirmed well-characterized markers for each cluster (Fig. 1g–i and Supplementary Table 4), such as high expression of AVP in hematopoietic stem cell (HSC) populations (Fig. 1g) and PF4 in megakaryocyte progenitors (Fig. 1h). Notably, CD271 enrichment facilitated clear identification of six distinct clusters from 5,301 cells, including osteoblasts and osteoclasts and endothelial and stromal vascular cells (Fig. 1i).

### An optimized antibody panel for human bone marrow analysis

**Fig. 2:**
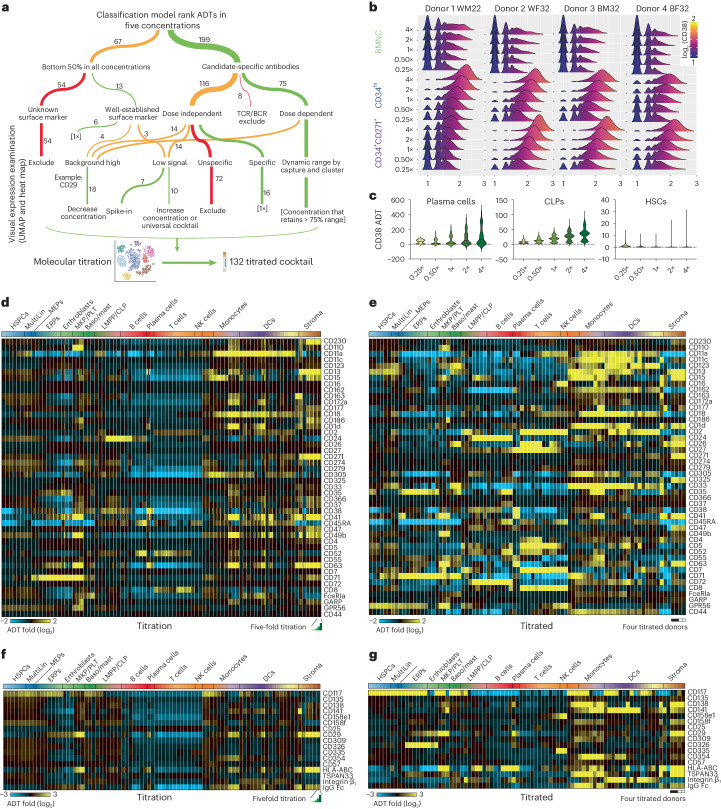
An optimized antibody cocktail for bone marrow progenitor characterization. **a**, Decision tree to nominate ADTs and optimal working concentration; TCR, T cell antigen receptor; BCR, B cell receptor. **b**, Distribution of CD38 ADT abundance (*x* axis) with changes in titration by capture and concentration (*y* axis). **c**, Cell population-specific detection of CD38 by concentration. **d**–**g**, Heat map of ADT expression by cell state and concentration for ADTs with improved detection in the 132 titrated cocktail (**e** and **g**) versus the titrations (**d** and **f**). ADTs for which concentrations were increased (**d** and **e**) or decreased (**f** and **g**) in the titrated mix are shown. Each cluster column of titration data (**d** and **f**) consists of five columns representing 0.25× to 4× relative to the ‘working’ concentration in the 275-plex titration mix. Each cluster column of titrated data (**e** and **g**) consists of four columns representing the donors in the order of African ancestry female, African ancestry male, European ancestry female and European ancestry male, respectively; Baso/mast, basophil/mast cell; PLT, platelets.

**Extended Data Fig. 3:**
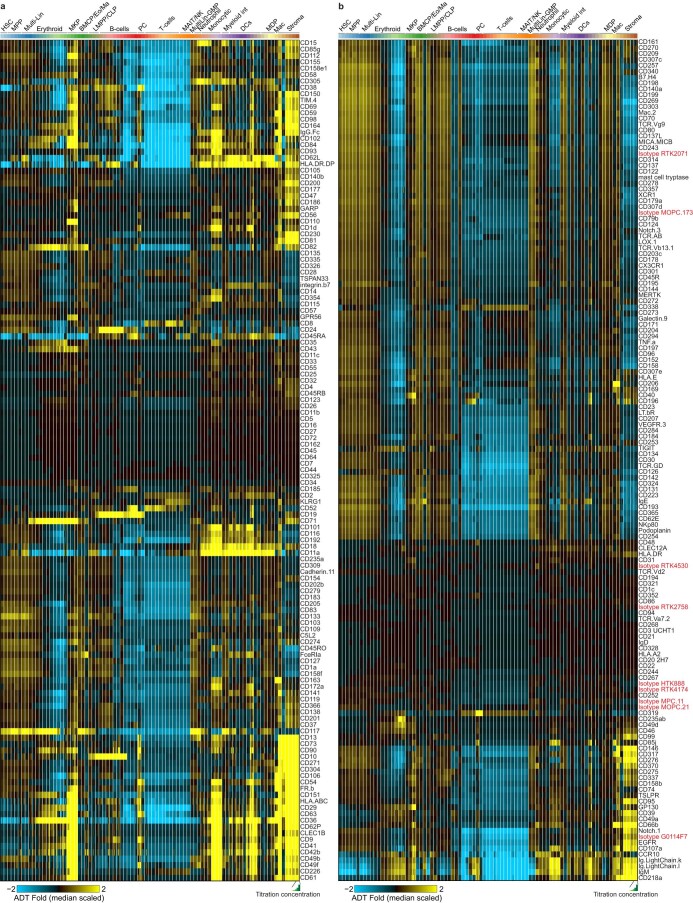
Heatmaps of all ADTs in the optimized titration atlas. **a**, Heatmap of 132 ADTs in the titrated CITE-Seq ADT mix. **b**, Heatmap of ADTs excluded and isotypes controls (highlighted in red).

**Extended Data Fig. 4:**
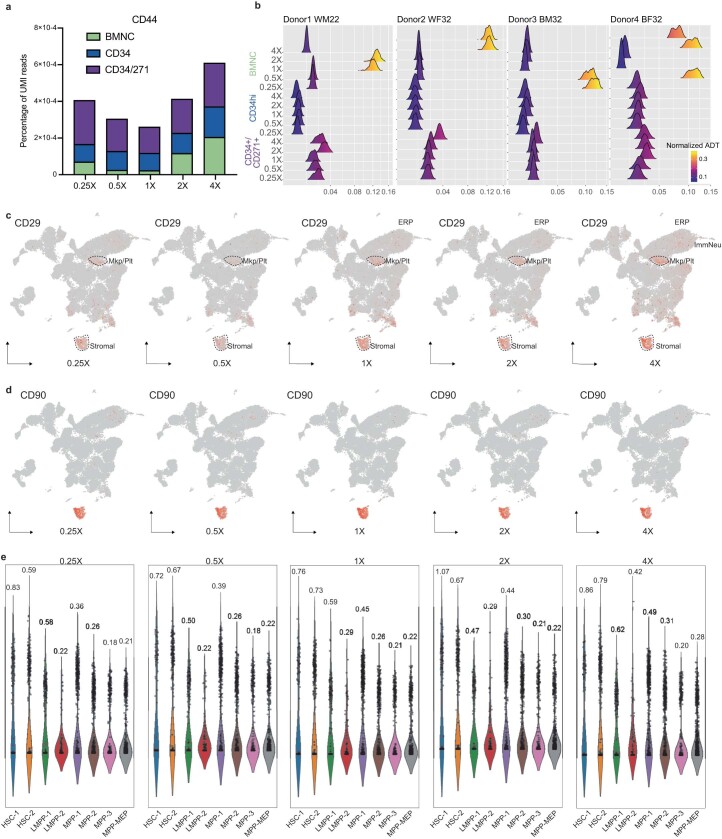
Additional examples of ADT concentration optimization. **a**, Percentage of CD44 ADT UMI counts in cells by capture and concentration. **b**, Ridgeplot examination of CD44 dynamic range by capture. Row heights vary based on expression. **c**, Specificity of CD29 ADT evaluated on UMAP at five titration concentrations. **d**, Specificity of CD90 ADT evaluated by UMAP visualization at five titration concentrations. **e**, Violin plots of CD90 ADT expression in stem cell populations. Mean shown on top of each plot.

The delineation of discrete and transitional transcriptomic cell states serves as the basis to define optimal ADT concentrations that selectively resolve these distinct populations. Optimal concentrations are needed to improve target detection, reduce nonspecific binding and minimize signal saturation of specific antibodies. Because there is no established protocol to evaluate antibody–ADT titration specificity, we applied a hybrid approach where ADT specificity was assessed by machine learning in conjunction with a multitiered threshold approach (Fig. 2a). To select a final list of ADTs, we applied decision trees and a gradient boosting approach (XGBoost^26^) to rank ADTs at each concentration based on their ability to separate the transcriptome-defined clusters following denoising and normalization (TotalVI^27^; Supplementary Table 5). Predictions were confirmed by directly assessing population specificity in our dataset relative to the literature, excluding nonspecific or lineage-irrelevant ADTs (Extended Data Fig. 3a,b and Methods). This resulted in a titrated cocktail set of 132 surface marker-targeting antibodies (125 lyophilized and 7 fresh spike-in, Supplementary Table 6). Notably, several standard hematopoietic lineage antibodies displayed low relative performance in the prototype PBMC and titration mixes (perhaps because of lyophilization). Thus, seven antibodies (‘spike-in’; CD34, CD16, CD4, CD90, CD45, CD11b and CD127) were included as a secondary step to improve detection of these antigens and eliminate confounding variables. Optimal concentrations for each ADT were determined using ridge plot visualization of raw and normalized counts (Methods), such as CD38 (Fig. 2b,c), CD44, CD29 and CD90 (Extended Data Fig. 4).

**Extended Data Fig. 5:**
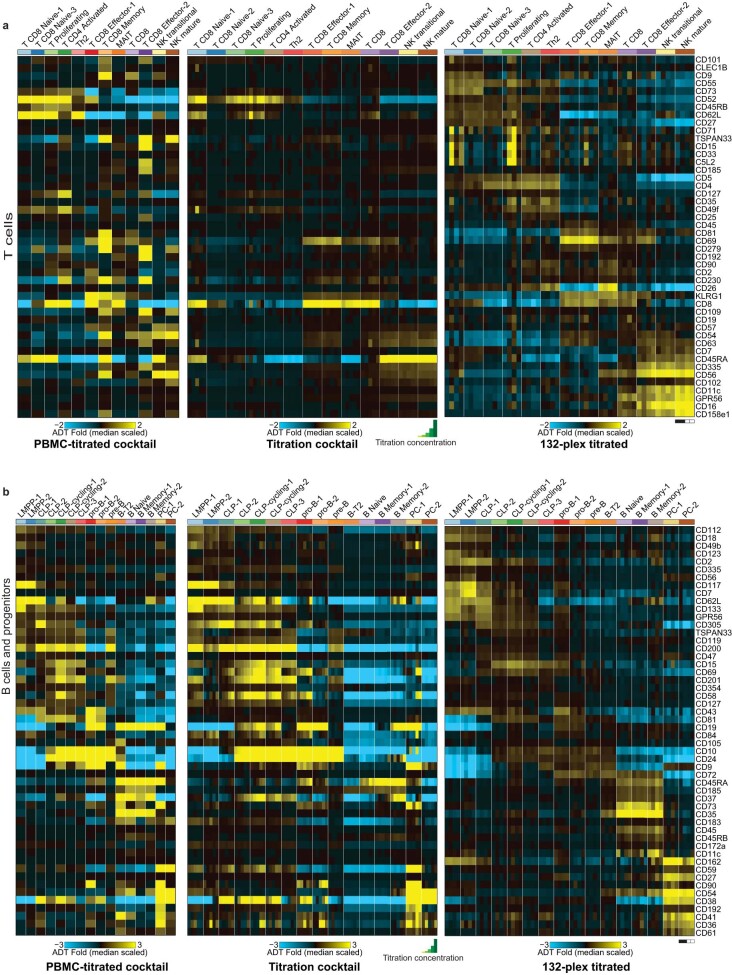
ADT marker specificity in lymphoid lineages. Top ADT markers examined in the PBMC-titrated cocktail (left, average expression of four titration donors), customized titration cocktail (middle, average expression of four titration donors at each concentration from low to high) and 132-plex titrated cocktail (right, average expression of each titrated donors, color decodes race) in T (**a**), B (**b**).

**Extended Data Fig. 6:**
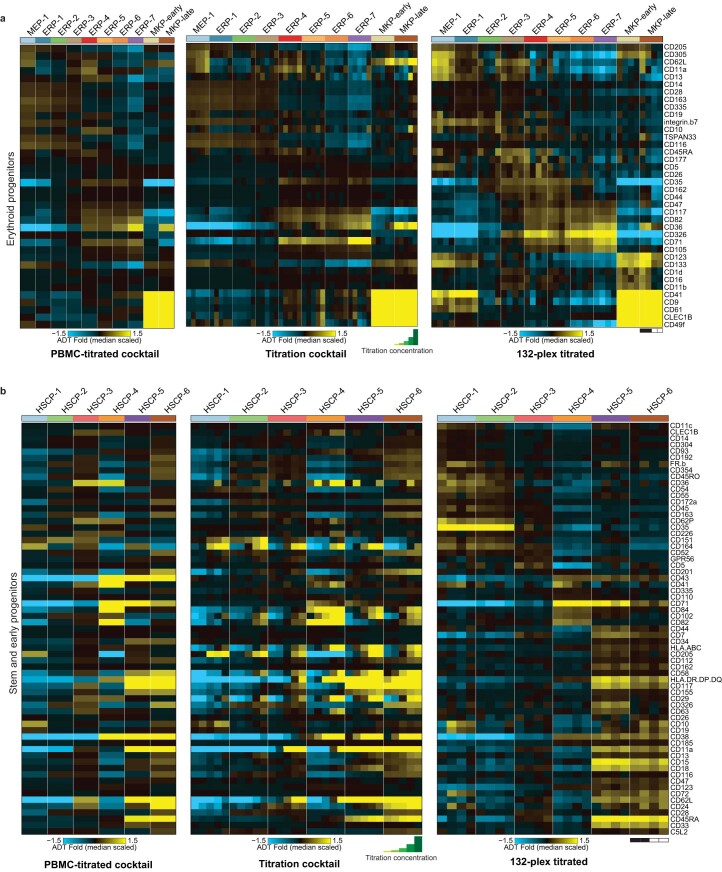
ADT marker specificity in stem and progenitors. Heatmap of the top ADT markers in erythroid/megakaryocytic (**a**) lineages and HSPCs (**b**).

To test the staining performance of the chosen antibodies, we applied the newly defined titrated CITE-seq panel to four new donors (using the same three populations as described above; BMNCs, CD34^hi^ cells and CD34^+^CD271^+^ cells). As anticipated, none of the ADTs occupied excessive sequencing space (>10%; Supplementary Table 7). Indeed, the titrated ADTs displayed consistently improved performance over the original titration likely due to more balanced proportions of each antibody, often resulting in cell population specificity not observed in the separate titrations (Fig. 2d–g and Extended Data Figs. 5 and 6). For example, the titrated mix enabled robust distinction among lymphocytes through known markers CD4, CD8 and CD19 (Extended Data Fig. 5) and early stem and progenitor cell populations through CD105, CD4 and CD304, which the original PBMC-titrated mix was unable to resolve (Extended Data Fig. 6).

### Multimodal analysis to resolve transitional cell states

**Fig. 3:**
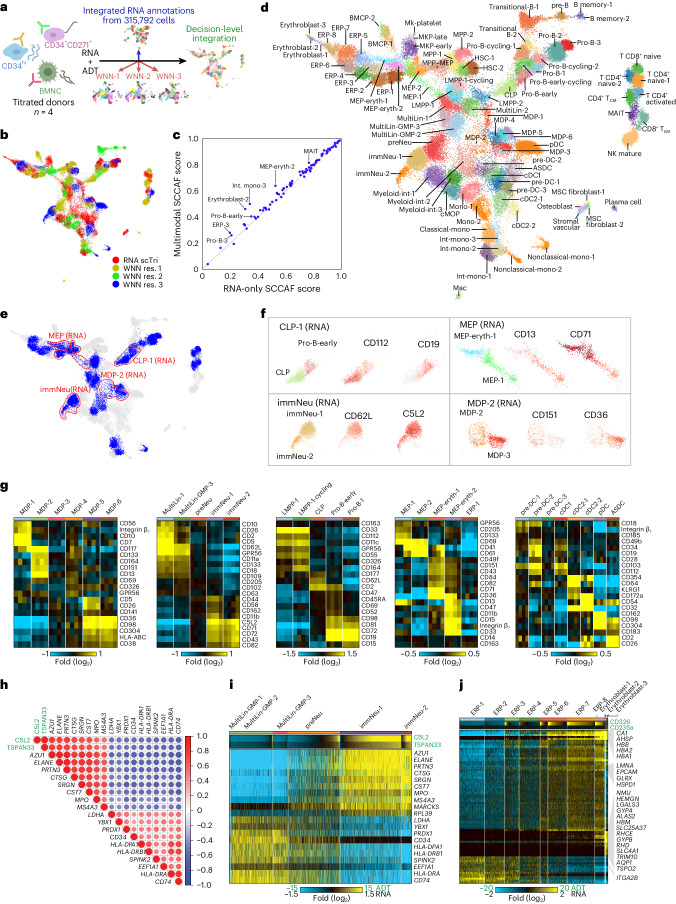
A multimodal progenitor cell atlas links surface markers with lineage specification. **a**, Schematic showing the multimodal cluster integration strategy from multiple clustering resolutions (MUON WNN) and transcriptome-defined cell states (scTriangulate). **b**, The most stable clusters defined by annotation source in the final scTriangulate (scTri) integration. **c**, Reclassification accuracy (SCCAF statistic) for the final scTriangulate clusters with (multimodal) and without considering ADT (RNA only). **d**, UMAP projection and annotation of the final 89 scTriangulate clusters. **e**, UMAP indicating multimodal scTriangulate clusters that were further separated with the addition of ADTs versus transcriptome-only clustering (see Fig. 1c). ‘Split’ clusters are indicated in blue, and populations outlined in red correspond to those in **f**. **f**, UMAP of example cell populations selectively resolved by the addition of ADTs. Exemplar ADTs are shown; red, relative expression; gray, no expression. **g**, Heat map of distinguishing ADT markers (MarkerFinder algorithm) for selected predicted lineage subsets. **h**, Pearson pairwise gene and ADT correlation similarity matrix dot plot for the top correlated and anticorrelated genes with C5L2 and TSPAN33 ADT expression among GMPs, preNeu and immNeus; green, ADTs. **i**, Heat map showing the relative abundance of C5L2 and TSPAN33 in granulocyte progenitors and correlated marker gene relative expression (median normalized). **j**, Heat map showing the relative abundance of CD326 and CD235a in ERPs and correlated marker gene relative expression (median normalized); eryth, erythroid; cMOP, common monocyte progenitor; cDC, classical DC. Source data

**Extended Data Fig. 7:**
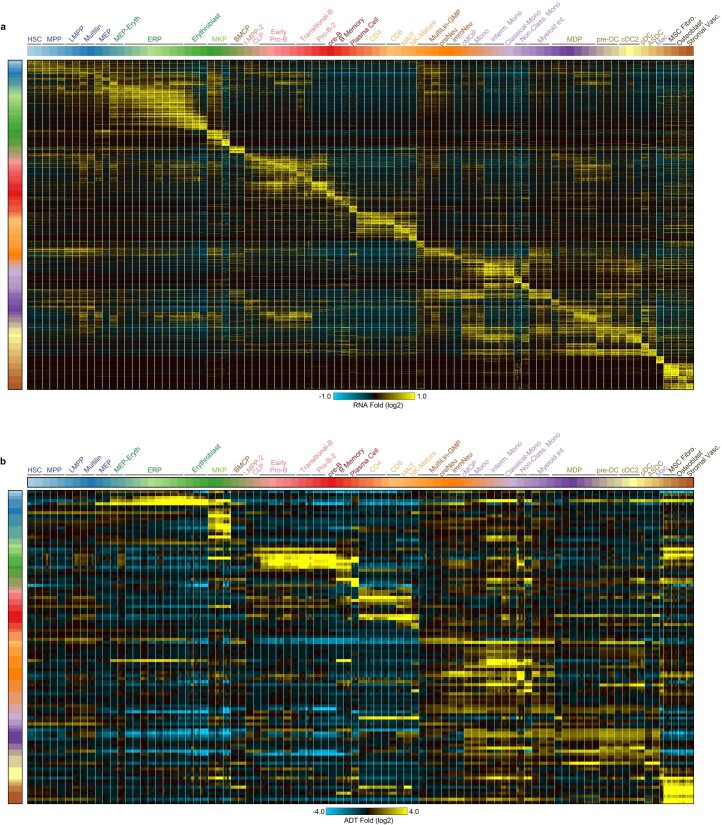
CITE-Seq titrated cell populations are defined by unique mRNA and ADT markers. Heatmap of the top 60 transcriptome (**a**) or top 5 surface epitope (**b**) markers from the software AltAnalyze (MarkerFinder algorithm) for all scTriangulate annotated clusters. Source data

Using this optimized CITE-seq dataset of 72,179 cells, we rederived clusters through a multimodal integrative strategy leveraging the Leiden weighted nearest neighbor (WNN) framework in ‘multimodal omics analysis’ (MUON)^28^. Specifically, low-, medium- and high-resolution WNN clusters, informed by both RNA and ADTs, were derived and integrated with RNA-based clusters from our initial single-cell compendium, again using scTriangulate (Fig. 3a). The most stable integrated clusters were a mix of those predicted by multiple WNN resolutions or RNA-based annotations (Fig. 3b). To quantify the value gained through the inclusion of titrated ADTs, we compared the stability associated with each cluster with and without ADT features measured by the multivariate single-cell clustering assessment framework^29^ (SCCAF) reimplemented in scTriangulate. SCCAF score is suitable to capture subtle and gradient changes due to its consideration of all features instead of a subset of markers. The addition of ADTs improved the stability of select cell populations, notably, intermediate monocyte progenitors, early ERPs and pro-B and mucosal-associated invariant T cells (Fig. 3c). These final 89 scTriangulate clusters were then annotated based on the original titration transcriptome clusters and surface protein expression (Fig. 3d and Supplementary Tables 8 and 9). As expected, well-established surface markers such as CD14/CD16/CD11c and CD4/CD8/CD45RA/CD45RO defined mature cell populations such as monocytes and T cells into classical/nonclassical/intermediate monocytes and CD4^+^ or CD8^+^ naive or activated T cells, respectively. Although several smaller cell populations (such as stromal and T cell subsets) could be identified in the larger initial transcriptomes from the CITE-seq titration dataset of 315,792 cells, these were lost in the smaller titrated CITE-seq dataset. However, other new populations, such as distinct megakaryocytic–erythroid progenitor (MEP), monocyte–dendritic cell progenitor (MDP) and monocyte and neutrophil subsets, were gained. Importantly, these analyses nominate numerous novel mRNA and ADT markers with relatively restricted expression across cell states (Extended Data Fig. 7 and Supplementary Tables 10 and 11).

Although the effect is minimized in the titrated CITE-seq panel, many ADTs were predicted to be stroma specific at a global level. This included the well-established human HSC marker CD90 (Thy-1), which is ~100-fold increased in detection in stromal cells versus in HSCs (Extended Data Fig. 4d,e). Thus, to demonstrate a clear demarcation of diverse cell lineage-predicted progenitor cell states by ADTs, similar to those observed by RNA (Figs. 1g–i and 3e), we next focused ADT analyses on presumptive hematopoietic lineage transitions. Overall, we found that ADTs were able to effectively split 16 of the original transcriptome-only defined clusters using label transfer (Fig. 3e,f). In all examined cases, we were able to identify ADTs that clearly demarcate the RNA + ADT integrated clustering. For example, the transition from lymphoid–myeloid primed progenitors (LMPPs) to early B cell progenitors was characterized by a transition from CD112^+^ (LMPP) to CD164^+^ (LMPP cycling), CD2^+^ (CLP), CD98^+^ (pro-B early) and CD19^+^CD15^+^ (pro-B-1) cells (Fig. 3f,g). Similarly, we observed coincident but low-level expression of megakaryocytic (CD41 and CD61) and erythroid (CD71 and CD82) markers in MEP-associated cell states.

To identify surface markers that reflect lineage specifications in hematopoiesis, we performed an ADT-to-gene correlation analysis. In granulopoiesis, multiple studies have shown discreet intermediate cell types, such as early neutrophil progenitors (Lin^–^CD66b^+/low^CD15^low^CD49d^+^CD11b^–^)^30^ and committed neutrophil progenitors (CD66b^–^CD64^dim^CD115^–^ in SSC^low^CD45^dim^CD34^+^ and CD34^low/–^)^31^; however these immunophenotyping strategies heavily rely on the exclusion of well-characterized markers from prior knowledge. By CITE-seq, we observed two novel surface markers C5L2 and TSPAN33, which were concordant (single-cell Pearson correlation) with the mRNA expression of well-described neutrophil maturation genes, such as *AZU1*, *ELANE*, *PRTN3* and *MPO*, and anticorrelated with the expression of primitive progenitor genes (for example, CD34, SPINK2 and *CD74*; Fig. 3h,i). These ADTs progressively increased in expression from presumptive granulocyte–monocyte progenitors (GMPs) to immature granulocytes (immNeu). In erythroblast differentiation, we also observed progressive induction of CD326 (*EPCAM*) surface expression in ERP-3 through ERP-8, whereas CD235a surface expression was progressively induced in later erythroblasts and later stages of erythroid differentiation. *EPCAM* was recently found to be expressed on erythroid-committed cells^23^. We further found that *EPCAM* expression is correlated with the induction of the extracellular secreted peptide neuromedin U (*NMU*), highlighting a potential association with niche signaling in erythropoiesis (Fig. 3j).

**Extended Data Fig. 8:**
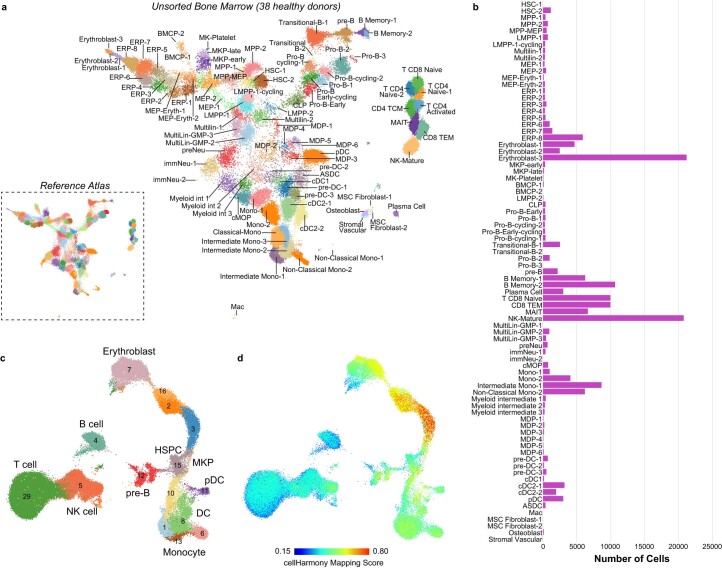
Validation of CITE-Seq evidenced cell populations in bone marrow biopsies from 38 donors. **a**, Projected UMAP of 242,832 healthy bone marrow mononuclear cells (10x Genomics Chromium platform) from the aggregate of three prior studies to our CITE-Seq fully titrated bone marrow reference. The reference CITE-Seq clusters used for label transfer and UMAP coordinate projection are shown in the inset. **b**, Bone marrow cell frequencies of all unsorted cells from 38 healthy donors. **c**, UMAP of unsupervised clustering (ICGS2) of cells from 20 bone marrow healthy donors (GSE120221). **d**, Projection of CITE-Seq label mapping scores from the software cellHarmony in the UMAP from panel **c** to infer annotation confidence.

Additionally, to ensure that our atlas is of sufficient comprehension and depth, we analyzed single-cell profiles from 38 prior published healthy bone marrow donors^3,20,32^. Alignment of these cells to our titrated scTriangulate clusters indicated representation of 88 of 89 cell populations (missing nonclassical monocyte-1); however, several populations had less than ten cells (immNeu-2, transitional B-2, BMCP-2 and macrophages; Extended Data Fig. 8a,b and Supplementary Table 12). As these low-abundance populations were evidenced by all CITE-seq donors in our cohort, we conclude that they are not artifacts. No clear additional cell populations were identified in this expanded dataset based on unsupervised analyses or cellHarmony mapping scores (Extended Data Fig. 8c,d). Notably, only 70 HSC-1 (0.04%) cells were identified in all 38 donors (*n* = 242,832), representing less than 3% of all presumptive HSPCs, whereas 1,832 HSC-1 cells were identified in our combined progenitor-enriched CITE-seq compendium (*n* = 406,681).

### Infinity Flow analysis to guide surface marker validation

**Fig. 4:**
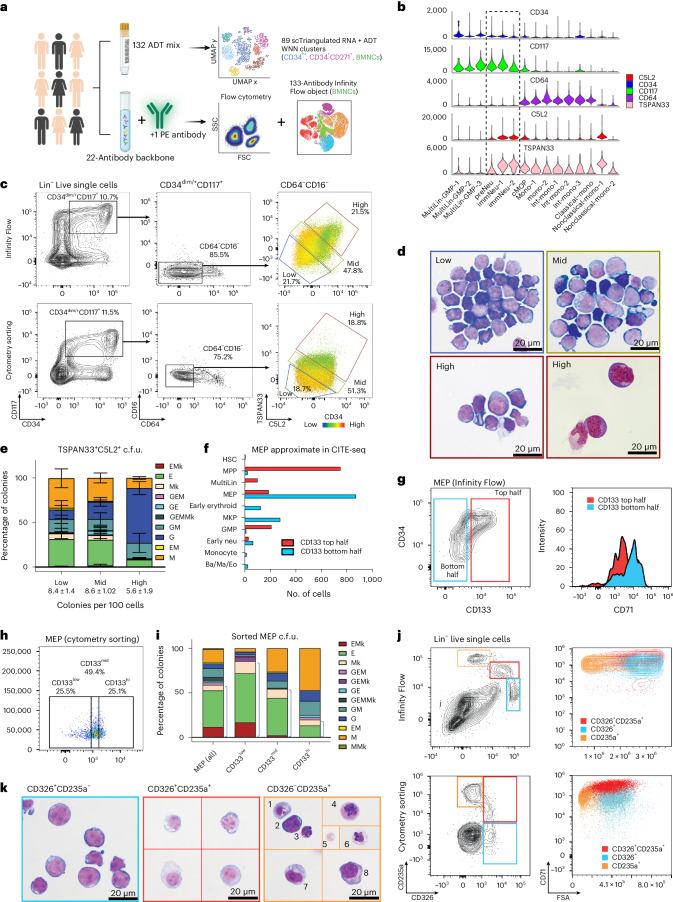
Identification of markers to dissect transitional cell states. **a**, Illustration of the donor cohort and Infinity Flow experimental design. **b**, Expression of surface markers that could enable purification of committed granulocyte progenitors. **c**, Proposed gating for C5L2^+^TSPAN33^+^ cells in Infinity Flow (top) and representative cytometry sorting (bottom). **d**, Cytospin morphology of cells sorted based on C5L2^+^TSPAN33^+^ expression, Wright–Giemsa staining of low (top left), mid (top right) and high (bottom left) and Quick III staining of high (bottom right). **e**, Differentiation potential of C5L2^+^TSPAN33^+^ sorted cells by c.f.u. assay. Data are shown as mean ± s.d. **f**, Cell composition in a virtually gated MEP subset by CD133 expression in CITE-seq data; Ba/Ma/Eo, basophil/mast cell/eosinophil; Neu, neutrophil. **g**, CD71 expression in immunophenotypically defined MEPs by CD133 expression in Infinity Flow. **h**, MEP subset by CD133 expression in cytometry sorting. **i**, c.f.u. readout of MEP cells sorted based on CD133 expression from the donor in **h** (data from the other donor are shown in Extended Data Fig. 9h). Blue brackets highlight the proportion of megakaryocytic/erythroid single and bipotential colonies. **j**, CD326 and CD235a denote three distinct cell populations in Infinity Flow (top) and cytometry sorting (bottom) that are different in cell size and CD71 expression. **k**, Morphology of sorted cells based on CD326 and CD235a expression; CD326^+^CD235a^–^, proerythroblasts; CD326^+^CD235a^+^, basophilic erythroblasts; CD326^–^CD235a^+^, (1) reticulocyte, (2) polychromatic erythroblast, (3) orthochromatic erythroblast, (4) basophilic erythroblast, (5) mature RBC, (6) transitioning polychromatic-to-orthochromatic erythroblast, (7) orthochromatic erythroblast and (8) orthochromatic erythroblast. The Infinity Flow object shown is representative of donor WM29. The data from the c.f.u. assays are the results of three replicates from two donors either combined (**e**) or displayed individually (**i**). Data in **d** and **k** are representative of ten high-power fields (differential count in Extended Data Fig. 9); c.f.u. types: granulocytic (G), monocytic (M), erythroid (E) and megakaryocytic (Mk) cells. Source data

**Extended Data Fig. 9:**
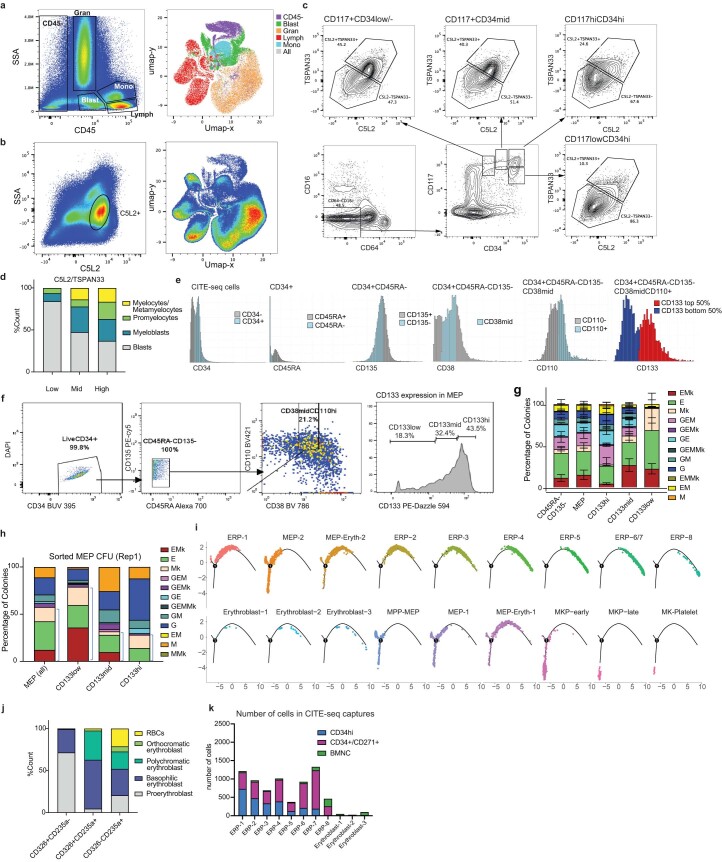
Validation of transitional cell states. **a**, Cells in a commonly used clinical diagnostic gate (CD45/SSA) and their presentation on UMAP. **b**, C5L2 + cells visualized on UMAP. **c**, CD34+/dim CD117+ cells examined for C5L2 and TSPAN33 expression. **d**, Cell count by morphology of C5L2/TSPAN33 Low/Mid/High sorted cells. **e**, Virtually gated ‘MEP’ in CITE-seq data. **f**, Sorting gate setup in CD133 index sorting. **g**, CFU readout of indexed sorted cells grouped by CD133 expression. Error bar shows Mean with SD. **h**, CFU readout of MEP cells sorted based CD133 expression from the other donor. **i**, Monocle trajectory analysis of erythroid progenitor hierarchy. **j**. Cell count by morphology in CD326+, CD326 + CD235a+ and CD235a+ sorted cells. **k**, Number of erythroid progenitors from each CITE-seq capture. Morphology cell counts are based on 10 high-power fields. CFU assays are results of three replicates from two donors either combined (**g**) or displayed individually (**h**). Source data

On their own, ADTs can nominate new cell isolation strategies but do not denote discrete flow cytometry solutions. Conventional flow cytometry is limited in the number of surface markers for a single cell. A recently developed protocol called Infinity Flow overcomes this limitation by imputing missing protein abundance values through machine learning with overlapping flow cytometry panels, and our Python version facilitates analysis of millions of cells^17,18^. To determine the validity of our CITE-seq ADT predictions, we generated matched Infinity Flow and CITE-seq data for the same 132 surface antibodies (clone matched) in a set of four donors. An additional five donors were processed for Infinity Flow with this set of antibodies (Supplementary Table 6). For Infinity Flow, multiple independent flow assays were performed on cells from a single sample, with each assay sharing a common set of 22-color backbone markers (modified from Cytek’s 20-color acute myeloid leukemia (AML) panel), with a different query fluorochrome-conjugated antibody (for example, phycoerythrin (PE)) added (*n* = 111). The cellular intensity of each additional marker was imputed using the optimized Infinity Flow machine learning workflow pyInfinityFlow^18^ (Fig. 4a). The resulting Infinity Flow data were initially annotated by lineage markers and side scatter properties (Extended Data Fig. 9a). As expected, we observed an enrichment in granulocytes in Infinity Flow relative to CITE-seq. Granulocytes were not efficiently captured on 10x platforms or were missed in cell calling. In agreement with this, the majority of C5L2 single-positive cells in total BMNCs were found to be mature granulocytes (Extended Data Fig. 9b).

Our CITE-seq analysis nominated antibody combinations predicted to define transitional cell states. To test these predictions, we confirmed that presumptive granulocyte progenitor/precursor populations ‘preNeu’, ‘immNeu-1’ and ‘immNeu-2’ cells gradually lose CD34 but maintain high expression of CD117 compared to MultiLin-GMP populations (Fig. 4b) and then applied conventional flow cytometry gating criteria to the Infinity Flow objects. Consistent with our CITE-seq results, C5L2 and TSPAN33 were coexpressed in multipotent (CD117^hi^CD34^hi^) and myeloid progenitors/precursors (CD117^+^CD34^mid^CD117^+^CD34^low/–^) but not in CD117^low^CD34^hi^ cells, with increasing C5L2^+^TSPAN33^+^ cells as CD34 expression decreased (Extended Data Fig. 9c). Of note, while these new markers (C5L2 and TSPAN33) also mark monocyte progenitors, we excluded monocytes by selecting for CD16^–^CD64^–^CD117^+^CD34^+^ cells. To verify the fate and clonogenicity of cells expressing C5L2 and TSPAN33, we prospectively sorted C5L2^hi^TSPAN33^hi^ (‘high’; ~20%), mid (~40%) and low cells (~20%) following the above progenitor gate. Consistent with CITE-seq and Infinity Flow predictions, the expression of these two markers was anticorrelated to CD34 expression (Fig. 4c). Cytospin morphology further confirmed that C5L2^hi^TSPAN33^hi^ cells are enriched for granule-containing granulocyte progenitors/precursors (Fig. 4d and Extended Data Fig. 9d). Colony formation assays indicated that C5L2^hi^TSPAN33^hi^ cells have low clonogenicity, with a small percent forming predominately granulocyte-only colonies (Fig. 4e). Conversely, C5L2^mid^TSPAN33^mid^ and C5L2^low^TSPAN33^low^ cells showed little cytoplasm and were multipotent. Hence, C5L2 and TSPAN33 appear to be novel markers for granulocyte commitment.

Currently, the best enrichment strategy for MEPs (CD34^+^CD38^mid^CD45RA^–^CD135^–^CD110^+^) still has contamination with granulocyte–monocyte colony-forming units (c.f.u.). When we virtually replicated cytometry gating of MEPs in CITE-seq (Extended Data Fig. 9e), we observed the presence of multipotent progenitors and MultiLin-GMPs, which are marked by CD133 expression (Fig. 4f). Infinity Flow objects revealed that CD133 expression bifurcated MEPs, with lower-CD133-expressing cells having higher CD71 expression (Fig. 4g). We then performed c.f.u. assays and index sorting to examine cell fate within the MEP gate by CD133 expression (Fig. 4h and Extended Data Fig. 9f). CD133^hi^ MEPs retained substantial granulocyte–monocytic potential, whereas CD133^low^ MEPs were enriched for megakaryocytic–erythroid potential (Fig. 4i and Extended Data Fig. 9g,h). Thus, we demonstrated that CD133 surface expression can be used to separate other-lineage progenitors from functionally bipotent MEPs.

In addition to distinct MEP subsets, our CITE-seq analyses identified a diverse spectrum of predicted ERP and erythroblast cell states. These included cell states defined by markers such as CD326 (*EPCAM*) and CD235a that are transiently expressed during sequential phases of erythroid differentiation. Stages of erythroid differentiation are traditionally denoted by changes in cell size and granularity (forward and side scatter), which can be assessed by flow cytometry but not CITE-seq. To examine the utility of the combination of CD326 and CD235a for dissecting late erythropoiesis (Fig. 3j and Extended Data Fig. 9i), we examined their expression and forward scatter in CD71^+^ cells (excluding mature red blood cells (RBCs)). As predicted, these two markers clearly separated three cell populations as CD326^+^CD235a^–^, CD326^+^CD235a^+^ and CD326^–^CD235a^+^ that are distinct in size and CD71 expression in both Infinity Flow and cytometry sorting (Fig. 4j). Cytospin morphology confirmed that CD326^+^CD235a^–^ cells consisted of proerythroblasts that are large with little cytoplasm, CD326^+^CD235a^+^ cells are a mixture of proerythroblasts and erythroblasts with high nuclear-to-cytoplasmic ratio and basophilic cytoplasm, and CD326^–^CD235a^+^ cells are generally smaller chromatin-condensed polychromatophilic erythroblasts, orthochromatic erythroblasts (normoblasts) and mature enucleated RBCs (Fig. 4k and Extended Data Fig. 9j). These findings demonstrate a progression from CD326^+^CD325a^–^ proerythroblasts (ERP-7/ERP-8; Extended Data Fig. 9k) and CD326^+^CD235^+^ maturing erythroblasts to CD326^–^CD235a^+^ late erythroblasts and nucleated RBCs that allows for a more granular refined strategy for staging of human erythropoiesis.

### Technology-agnostic stable markers for lineage identity

**Fig. 5:**
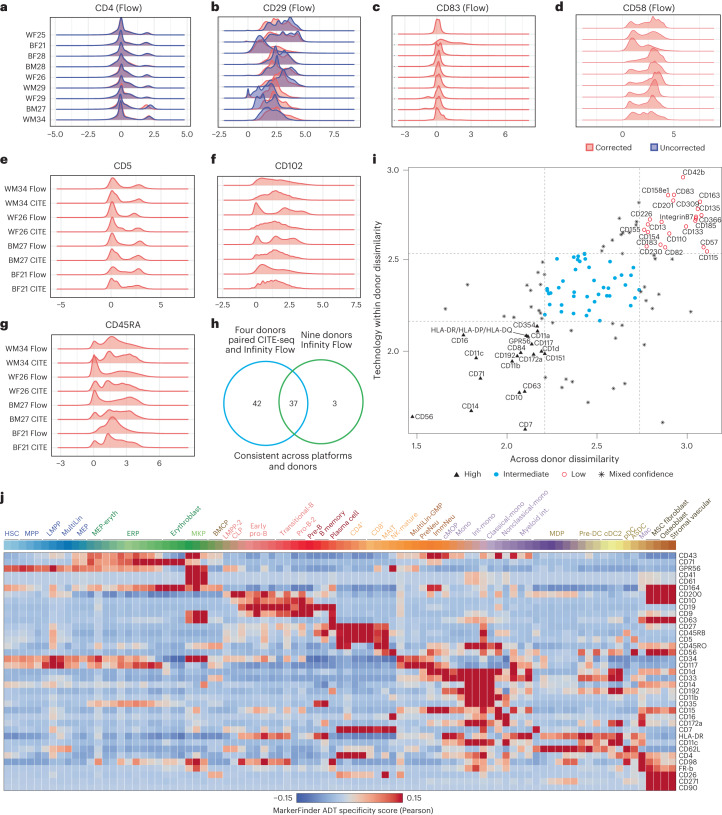
Variation of surface marker expression across donors and between technologies. **a**, Consistent expression pattern of CD4 presented in a ridge plot before and after correction in nine donors. **b**, Inconsistent expression of CD29 presented in a ridge plot before and after correction in nine donors. **c**, CD83 corrected expression presented in a ridge plot is consistent across eight donors. **d**, CD58 corrected expression presented in a ridge plot is inconsistent across donors. **e**, Consistent expression of CD5 across technologies and donors. **f**,**g**, Varied expression distribution in both donors and across technologies (CD102 (**f**) and CD45RA (**g**)). **h**, Venn diagram showing surface markers that exhibit consistent expression across donors and technologies by ridge plot distribution visualization. **i**, Surface marker expression dissimilarity examined by Anderson–Darling testing. Across donor (*x* axis) and technology within donor (*y* axis) distribution dissimilarity for *n* = 132 markers (see Methods). Dissimilarity values are the log_10_-transformed values of the Anderson–Darling test *t* values. **j**, Heat map showing the correlation of 37 highly consistent surface markers from **h** with multimodal clusters from titrated data. The batch effect correction was performed with cyCombine; pDC, plasmacytoid DC. Source data

**Extended Data Fig. 10:**
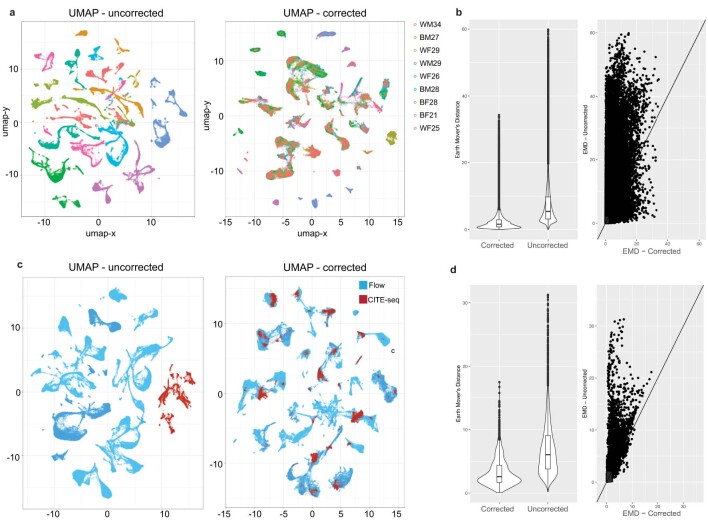
Integration of global CITE-Seq and Infinity Flow antibody profiles. **a**, UMAP representation of Infinity Flow objects from each of the 9 different analyzed donors. **b**, Earth Mover’s Distance (EMD) dissimilarity evaluation of correction within the Infinity Flow data to assess variance between donors (n = 9). **c**, UMAP indicating the integration of 4 matched CITE-seq and Infinity Flow datasets (n = 4). **d**, EMD evaluation of correction between CITE-Seq and Infinity Flow for the four matching sample pairs. The Violin plots show the inter-quartile range and outlier cells from the indicated EMD distributions.

To establish direct links between specific markers in the CITE-seq and Infinity Flow data, we looked to co-normalize these two datasets. This analysis requires that measured cell surface markers have similar intensity distributions across technologies and donors. The distribution of antibody signal intensities is typically unimodal or multimodal (peaks) across all cells, reflecting distinct cell populations and surface marker expression range. As our collection of donors varied by both race and sex, it is unclear which antibodies will demonstrate consistent profiles across donors. Indeed, we observed substantial variability in the distribution of signal intensities of antibodies across donors (Extended Data Fig. 10a). To minimize variability due to technical batch effects, we turned to a recently developed empirical Bayes batch correction protocol called cyCombine^33^ integration. After applying cyCombine, we observed significant adjustment in the antibody intensity values (Earth Mover’s Distance dissimilarity index of 0.69; Extended Data Fig. 10b). Although certain markers, such as CD4, showed minimal alterations after correction (Fig. 5a), others, such as CD29, underwent substantial changes in their expression profiles (Fig. 5b). The distribution of antibodies further varied among donors for a high proportion of the 132 markers after correction. For example, CD83 exhibited a unimodal distribution in six donors and a multimodal distribution in the remaining three donors (Fig. 5c), whereas CD58 manifested two prominent peaks in four donors, with varying peak configurations observed in other donor samples (Fig. 5d).

To define the antibodies that perform equally well across donors and technologies, we next attempted to integrate batch-corrected antibody profiles between CITE-seq and Infinity Flow data from the four donors profiled. For these analyses, we applied a statistical testing procedure of inferred antibody abundance (*k*-sample Anderson–Darling test) as well as expert-based curation. Before correction, technology-associated batch represents the major source of variation within the integration (Extended Data Fig. 10c). However, after correction, we obtained a joint integration for individual donors (Earth Mover’s Distance = 0.54; Extended Data Fig. 10d). Expert curation of all surface markers following cyCombine nominated 79 antibodies consistent between CITE-seq and Infinity Flow (for example, CD5), with the remainder exhibiting inconsistency between platforms in their modal distribution (for example, CD102 and CD45RA; Fig. 5e–g). By contrast, we found variability in antibody consistency when only considering Infinity Flow across the nine donors, with only 37 markers consistent across all samples (Fig. 5h and Supplementary Table 16). Application of the *k*-sample Anderson–Darling test additionally found significant variation in the consistency of markers, with a general consensus for the top performers by both *k*-sample Anderson–Darling test and expert curation (Fig. 5i and Supplementary Tables 16 and 17). The most consistent markers spanned both mature cell types and progenitors (Fig. 5j). Thus, our analyses nominate dozens of highly consistent cell surface markers that can be integrated across technologies and donors.

### Defining the origins of malignant cell states in leukemia

**Fig. 6:**
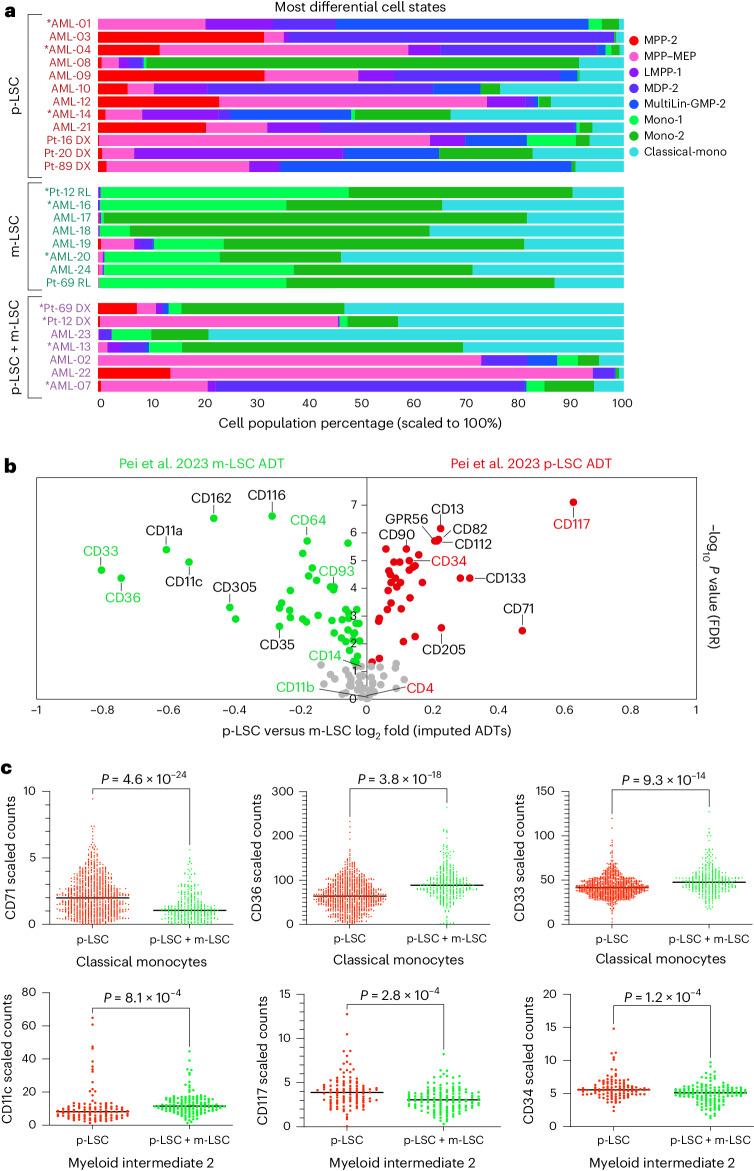
AML LSCs align with distinct healthy multilineage progenitors. CITE-seq bone marrow biopsies from individuals with AML with prior evidence of p-LSCs (sensitive to venetoclax/azacytidine), m-LSCs (resistant to venetoclax/azacytidine) or both (p-LSCs + m-LSCs) aligned to our multimodal atlas (Azimuth). **a**, Cell frequency was compared between AML captures. Asterisks (*) indicate that the sample LSC type was validated by xenograft. **b**, Differences in imputed cell surface ADT abundance (Azimuth) comparing p-LSC to m-LSC AML CITE-seq biopsies. Corresponding differentially expressed ADTs from the original study CITE-seq datasets^34^ are indicated in green (m-LSC enriched) or red (p-LSC enriched). **c**, Example of differentially expressed surface markers in p-LSC (*n* = 2) versus p-LSC + m-LSC (*n* = 1) samples for ADT TotalVI scaled counts within the indicated mapped cell states. Dots represent individual cells. Displayed *P* values were computed from a two-sided empirical Bayes moderated *t*-test (FDR corrected). Source data

Optimal stem cell and progenitor isolation strategies have important implications for marrow transplantation and monitoring of clonal cell frequencies in malignancy. Thus, beyond the identification of new cell states, clarifying antibodies and new flow cytometry strategies, our human marrow progenitor atlas presents the opportunity to resolve unexplored cellular relationships in hematological disease. In AML, treatment response has been related to heterogeneity within samples, in particular leukemic stem cell (LSC) heterogeneity. For example, venotoclax-resistant AML can in some instances arise from a monocytic LSC (m-LSC) as opposed to a more primitive LSC (p-LSC) with a distinct bulk immunophenotype (CD4^+^CD34^–^CD11b^–^CD14^–^CD36^–^)^34,35^. In these studies by Pei et al., xenograft assays were exploited to confirm LSC activity and differential sensitivity of p-LSCs and m-LSCs to therapy^34,35^. In malignant disease, hematopoietic cell identity is highly perturbed, resulting in cell programs that vary by individual, genetics and treatment stage, making integrated unsupervised analyses of single-cell genomics challenging^32^. To determine whether our marrow progenitor cell atlas could resolve these distinct AML LSC subsets and infer their likely normal stem and progenitor cellular origins, we aligned prior CITE-seq profiles from biopsies from individuals newly diagnosed with AML or those who received a diagnosis of AML relapse^34^. To enable direct comparison of these malignant profiles to our healthy multimodal references, we developed and deployed a hosted Azimuth Shiny server for broad community reuse (https://altanalyze.org/MarrowAtlas/). Alignment of the published AML scRNA-seq profiles with this Azimuth browser identified substantial differences in progenitor cell frequency versus that observed in healthy donors (Supplementary Table 18). Beyond differences versus healthy individuals, comparison of differential population frequencies in m-LSC-only versus p-LSC-only AML identified 14 highly varying cell states (*t*-test, *P* < 0.05). Not surprisingly, m-LSC AML was enriched in monocyte and DC progenitors (monocyte-1, monocyte-2 and classical monocytes), whereas p-LSC AML was most significantly enriched in our predicted LMPP-1 along with other stem and progenitor populations (MPP-2, MDP-2, MultiLin-GMP-2; Fig. 6a).

Improved flow cytometric gating approaches could enable real-time monitoring of LSC immunophenotypes before and during therapy. Although the study by Pei et al. used a limited CITE-seq panel (*n* = 22), only a few antibodies were able to differentially resolve p-LSCs and m-LSCs. Thus, we asked if our normal bone marrow atlas reference could predict new LSC-distinguishing antibodies and resolve their cells of origin. Applying the default Azimuth ADT imputation function to all 12 predicted p-LSC and 8 m-LSC prior AML CITE-seq captures, we were able to distinguish the most significant differentially expressed ADTs (Fig. 6b). This analysis produced ADT measurements that were concordant with the original limited CITE-seq analysis (Pearson *ρ* = 0.56), with the same top-predicted ADT markers for p-LSCs and m-LSCs at the sample level (Fig. 6b). Differentially abundant ADTs could be reflective of overall LSC lineage cell frequency or altered transcriptional programs within individual leukemia cell populations. For validation, we applied our optimized CITE-seq panel to three of the exact same leukemia samples from Pei et al. (two with functionally defined p-LSCs and one with p-LSCs + m-LSCs). Analysis of these 29,691 cells using our healthy reference cell atlas resolved 28 cell states detected in both p-LSC and p-LSC + m-LSC samples. To define ADTs that differ within the same cell state between p-LSC + m-LSC and p-LSC samples, we applied the cellHarmony differential expression analysis workflow. cellHarmony identified 69 antibodies within 23 cell states with at least 10% difference in abundance between p-LSCs and p-LSCs + m-LSCs (empirical Bayes *t*-test *P* < 0.05, false discovery rate (FDR) corrected; Supplementary Tables 18 and 19). Among these antibodies, we found that classical monocytes, myeloid intermediate-2 and B memory-1 cells with both m-LSCs and p-LSCs were also evidenced from our imputation analysis in Fig. 6b (Fig. 6c). These include antibodies originally reported by Pei et al. (for example, CD33 and CD64) in addition to new antibodies from our panel (for example, CD71). Thus, our data support the model in which distinct LSCs are partially defined by unique cell surface proteomes in addition to altered progenitor cell frequencies, contributing to clonal adaptation and survival. Such markers may represent attractive biomarkers for clinical monitoring beyond assessing lineage identity.

## Discussion

The isolation and characterization of primitive human hematopoietic cell states will enable the development of improved therapies for diverse malignant and nonmalignant diseases. Key to such insights are cell surface antibody cocktails that can be jointly applied in complementary high-throughput multimodal analyses of human primary bone marrow samples. We report a panel of 132 precision ADTs for prospective isolation and characterization of discrete bone marrow hematopoietic cell states using both CITE-seq and Infinity Flow. We believe our highly focused capture strategy of the earliest HSPCs, intermediate cell states plus stromal populations and the most abundant end states provides the deepest view of bone marrow stem and progenitor compartments described to date. Furthermore, relative to prior bone marrow cell annotation efforts, the combination of scRNA-seq and antibody staining resolved dozens of new cell states that are reproducible among donors with diverse ancestry and of either sex. Beyond normal hematopoiesis, these cell populations and CITE-seq cocktail informed the identification of discrete leukemic cell states and distinguishing surface markers associated with functionally defined p-LSCs and m-LSCs.

In this study, discrete cell states were resolved using new advances in machine learning and game theory (scTriangulate) that discriminate poorly resolved cellular intermediates from molecularly stable populations (marker gene/ADT and reclassification accuracy). The utility of our genomic–cytometric approach was exemplified by the identification and validation of novel surface antigen combinations for myeloid lineage specification (C5L2 and TSPAN33) and unique erythroid maturation stages (CD326 and CD235a) and an improved method for enriching MEPs using differential CD133 levels. Such transitional cell states can be selectively isolated and enriched to define populations with more restricted clonal outputs (MEPs) using CITE-seq-nominated markers. As such, these data strongly support a model in which discrete stem and progenitor cell states exist with restricted multi- or unipotentiality, as opposed to a model in which hematopoiesis is a continuum (which by definition lacks isolatable stable intermediate states such as MEPs).

Our atlas represents an essential starting point for the discovery of new cell surface antigens to distinguish and isolate discrete progenitor cell states. We provide these data as a reusable platform for cell-type annotation, cell surface antibody abundance imputation and exploration for the development of new progenitor isolation strategies. We expect these findings to fuel new insights into the signaling and transcriptional regulatory networks underlying hematopoiesis and improved flow cytometric markers to monitor malignant stem cells in disease.

Future studies will necessitate expanded cohorts of ethnically diverse healthy control individuals across the lifespan to understand the intrinsic and extrinsic variables that are associated with our observed variation. Moreover, such work will need to surmount numerous challenges for comparing and integrating digital antibody detection from approaches such as CITE-seq and fluorophore- and cytometry by time-of-flight-based detection approaches. Although our current study nominated dozens of markers consistent across technologies and donors, this number is likely an underestimate, as flow cytometry batch effect correction methods are still evolving, with larger evaluation cohorts required to understand dynamic variation. We are hopeful that new deep-learning integration models will resolve the discrepancies of surface marker detection between technologies and donors.

Similar to our exemplar study of AML LSCs, we expect that this digital multimodal bone marrow atlas will enable the discovery of cellular and molecular regulators of diverse hematopoietic diseases. Malignant hematopoiesis is frequently accompanied by dramatic changes in gene expression, disrupted cellular niches, altered cellular identity and infiltrating inflammatory regulators. Hence, our atlas can serve as a starting point for cell-type curation to identify normal cellular analogs that accrue mutations in malignancy but will need to be further integrated with new disease atlases to infer malignant lineage trajectories, missing cells and disease programs. To enable immediate re-classification and cell surface marker imputation from independent scRNA-seq datasets, we provide a dedicated Azimuth web interface for automated user queries. We further provide ShinyCell browser instances and donor-level RNA/ADT viewers, to explore and compare RNA/ADT marker expression, donor covariates, quality control, alternative cell annotation labels and different ADT titrations across the entirety of our reference CITE-seq compendium. These digital resources can facilitate new disease queries and lower the entry barrier for analysis from RNA alone.

Last, we expect the demonstrated analytical and experimental optimization strategies to guide future progenitor characterization strategies outside of hematopoiesis. This genomic–cytometric resource further lays the foundation for comprehensive, integrated analyses that bridge multiplexed sequencing and flow cytometry assays.

## Methods

This work complies with all relevant ethical regulations. Approval for the collection and analysis of adult healthy fresh human bone marrow aspirates and participant consent were obtained by Lonza. These samples were consented by the donors to share the age, sex and self-identified ancestry and raw sequencing data in an open manner. Sample size was determined based on availability of bone marrow donor ethnicity and sample volume, which is comparable to prior bone marrow atlas studies^20^. Samples were randomly selected for each experiment based on availability, and no donor was excluded. Sequencing data collection and analysis were not performed blind to the conditions of the experiments. Operators who performed morphology and c.f.u. evaluations were blinded of experiment group in validation experiments.

### Sample preparation for CITE-seq

For the TotalSeq-A titration experiment, 100 ml of fresh bone marrow from each healthy donor was purchased from Lonza and shipped overnight at 4 °C. All four donors were nonsmokers and tested negative for human immunodeficiency virus and hepatitis B and C viruses. For each donor, BMNCs were isolated by Ficoll Paque Plus (GE17-1440-02, Sigma-Aldrich) gradient centrifugation using SepMate-50 tubes (85450, STEMCELL Technologies). A small fraction of BMNCs was flow sorted using a Sony MA900 cell sorter (Sony Biotechnology) for live cells (7AAD^–^) and granulocyte depleted by side scatter for better BMNC capture quality. The rest of the BMNCs were stained using a Miltenyi CD34 indirect kit (130-046-701, Miltenyi Biotec) and were enriched for CD34^+^ cells (CD34^hi^) on an autoMACS separator (Miltenyi Biotec) using the Possel-d program setting. The negative fractions were stained with a CD271 MicroBead kit (human; 130-099-023, Miltenyi Biotec) and enriched on a Miltenyi autoMACS separator (program Possel) for coelution of CD271^+^ cells (CD34^+^ and/or CD271^+^). For the validation experiment and full-spectrum flow cytometry, 25 ml of fresh bone marrow samples was used. Cells were isolated as described above and split between CITE-seq and spectral flow cytometry workflows. Frozen total mononuclear cells from the bone marrow of three individuals with AML were sorted for live cells and processed using the same protocol as in the validation experiment.

Flow cytometry cell staining buffer consisting of DBPS (14-190-250, Thermo Fisher) with 2% fetal bovine serum (FB5002-H, Thomas Scientific) was used in the washing steps unless otherwise specified. Donor information can be found in Supplementary Table 1.

### TotalSeq-A antibodies

All TotalSeq-A antibody mixes are recommended for staining up to 500,000 cells in a volume of 50 μl. The 275-plex titration antibody cocktail (PN 900006213, BioLegend) was custom made based on TotalSeq-A Human Universal Cocktail, V1.0 (399907, BioLegend) and a prototype 277-plex human antibody cocktail (PN 900003129, BioLegend) that was titrated by BioLegend on PBMCs. We determined/verified the concentration of 47 antibodies in the titration cocktail (900006213, BioLegend) via three twofold dilutions using flow cytometry and hybridized oligo(dT)-Alexa Fluor 647 (/5Alex647N/TTT TTT TTT TTT TTT TTT TTT TTT TTT TTT, Integrated DNA Technologies) on human apheresis products. Concentrations of the other antibodies in 900006213 were determined based our previous experience with 900003129. When the amount of ADT occupied >10% of the total sequencing reads, the concentration was decreased. For ADTs that provided low sequencing reads, the concentration was increased. ADTs used in titration and validation experiments can be found in Supplementary Table 2.

### Single-cell CITE-seq generation

For the TotalSeq-A antibody titration experiment, CD34^hi^ and CD34^+^CD271^+^ cells were stained with unique TotalSeq-A anti-human Hashtag antibodies (Extended Data Fig. 1) to denote concentration and a previously sequentially diluted TotalSeq-A 275-plex antibody cocktail (4× to 0.25×) or a 277-plex antibody mix. BMNCs were stained similarly but without the 277-plex antibody mix. CD34^hi^ cells and CD34^+^CD271^+^ cells were washed on a Laminar Wash MINI System (Curiox Biosystems) with the following settings: 25 cycles, flow rate of 10 μl s^–1^ and initial volume of 55 μl. After washing, the CD34^hi^ and CD34^+^CD271^+^ cells were pooled per donor, whereas BMNCs were pooled by combining two donors. Of note, cells were stained and washed on a Laminar Wash 16-well plate in a staggered manner so that the staining step was timed for 30 min at 4 °C consistently across all populations/concentrations. Pooled cells were counted by Trypan Blue staining (15250061, Thermo Fisher Scientific) on a hemacytometer (3120, Hausser Scientific), and viability was over 90% before 10x chip loading. CD34^hi^ and CD34^+^CD271^+^ cells (97,000–115,000 per well) were loaded in eight wells using a 10x Chromium X 3′ version 3.1 HT kit (1000370, 10x Genomics), whereas BMNCs were loaded in four wells (16,000 per well) using a 10x Chromium standard 3′ version 3.1 kit (1000268, 10x Genomics). Emulsion, Gel Bead-In Emulsions (GEM) collection, clean-up and cDNA amplification with ADT/hashed tag oligonucleotide (HTO) spike-in primers were performed according to 10x Genomics and BioLegend TotalSeq-A protocols. Of note, HTO/ADT-containing fractions were further cleaned with 1.2× SPRI beads from 0.6× SPRI cDNA selection supernatant after cDNA amplification and were two-in-one pooled like cDNA in HT kits for HTO and ADT sample indexing PCR.

For the titrated CITE-seq donor samples, three populations from each donor were stained with the 132 titrated antibody cocktail including seven spike-in antibodies and washed by laminar flow. Cell number was counted, and populations were loaded in three ports without hashing (16,000 per well) with a 10x 3′ V3.1 kit. ADT libraries were amplified and cleaned similarly in step 2.3d where supernatant is separated. Clean-up and cDNA amplification were performed according to the standard TotalSeq-A protocol.

### Library preparation and sequencing

Library preparation was performed according to the manufacturer’s protocols. To account for the high number of cells from the HT kits, two to three cycles were reduced in most PCR amplification steps. Final transcriptome, ADT and HTO libraries were quantified and analyzed using a Qubit dsDNA HS assay kit (Q32854, Invitrogen), a High-Sensitivity DNA kit (5067-4626, Agilent Technologies) on a 2100 Bioanalyzer (G2939BA, Agilent Technologies) and a KAPA HiFi library quantification kit (KK4824, Roche). Dual-indexed transcriptome libraries from titration experiments were pooled and sequenced on two Illumina S4 flow cells with the PE150 + 10 + 10 setting (Illumina), and libraries from validation were sequenced across multiple S4 flow cells. Single indexed ADT and HTO libraries from titration experiments were pooled and sequenced on an Illumina S2 flow cell with the PE30 + 8 setting. ADTs from validation experiments were sequenced alone or with transcriptome libraries on S4 flow cells with PE100. BCL files were demultiplexed into fastq files for CellRanger input. ‘AT’ was added to the end of the RPI-x ADT i7 index (6 base pairs) to match the D70X_long HTO index (8 base pairs). HTO and ADT FASTQ files were supplied as 3P feature barcodes together with transcriptome FASTQ files into the Cell Ranger V6.1.2 count pipeline. The transcriptome was mapped to hg19 and hg38 reference genomes for downstream analysis and visualization. In total, 484,637 cells were recognized by Cell Ranger, with a median gene count ranging from 1,196 to 3,858 and a median ADT unique molecular identifier count ranging from 587 to 2,726 per cell.

### HTO calling and quality control

Cells were multiplexed using HTOs to distinguish both donor and CITE-seq ADT concentration. HTO barcode count matrices were obtained through the multimodal analysis workflow in Cell Ranger before normalization (counts per ten thousand (CPTT)). Cell barcodes with >30% of normalized reads assigned to multiple HTOs were annotated as doublets, with confident singlet predictions assigned to cells with >40% of normalized reads assigned to a single HTO (HTO processing module of AltAnalyze). Cells were further filtered based on the seven mouse/rat isotype control antibody counts (see the source code in Data availability) and performed quality control filtering in Seurat V4 (ref. ^36^) by nFeature_RNA > 500 & nCount_RNA > 1000 & percent.mt < 25. This quality control step filtered 393,748 cells to 315,792 high-quality single cells in the initial titration dataset and 90,889 to 72,198 cells in the final titrated CITE-seq dataset.

### CITE-seq analysis

All Cell Ranger-produced count matrices underwent ambient RNA exclusion using the software SoupX^37^ with a contamination fraction of 15% and quality control filtering by HTO and Seurat V4 (ref. ^36^). Ambient corrected transcriptome counts and associated ADT counts were supplied as input to the software TotalVI to obtain normalized and denoised ADT counts. To derive clusters from the initial titration CITE-seq datasets, the software cellHarmony was used to transfer labels from CPTT normalized expression centroids computed in author-provided labels from three prior published reference bone marrow atlases. Cell annotations from an integrated multicohort human bone marrow atlas from the Azimuth website (https://app.azimuth.hubmapconsortium.org/app/azimuth-bone-marrow) were projected onto the initial titration dataset using the default mapping function in the Azimuth R Shiny web interface (level 2 annotations). Unsupervised clustering was performed using a two-step process in the software ICGS2 using 5,000 cells used for PageRank downsampling and a minimum marker Pearson threshold of 0.2. For this analysis, SoupX-corrected CPTT expression files were combined and supplied to AltAnalyze version 2.1.4. This workflow automatically selects the optimal cluster resolution based on marker gene cluster filtering, ignoring nonrobust and doublet cell clusters. Cells with a poor mapping score to the final clusters (linear support vector classification coefficient > 0) were excluded from the analysis (for example, doublets). This analysis identified 33 initial clusters with no evident donor-specific effects. These clusters were grouped into seven broad lineage classes: HSPCs, early lymphoid and B cells, T and NK cells, stromal cells, myeloid cells, erythroblasts and basophil/mast cell progenitor/megakaryocyte progenitors. ICGS2 was rerun independently on all seven classes to produce a set of combined subclusters. All candidate supervised and unsupervised cluster annotations were provided as inputs for the software scTriangulate version 0.13.0 to identify the most stable integrated cluster annotations. To refine the final cell annotations and exclude putative doublet cell assignments, final cluster annotations were derived by remapping cell barcode transcriptome profiles to the scTriangulate cluster centroids following MarkerFinder feature selection on 50 representative cells per cluster. Three initial scTriangulate clusters out of the original 88 were excluded from this analysis due to low cellHarmony remapping scores (Pearson correlation < 0.5). These cell annotations were projected again with cellHarmony onto the final titrated CITE-seq dataset to derive initial transcriptome annotations. WNN^28^ was applied using three Leiden clustering resolutions (1, 2 and 3) in the MUON framework^28^ to obtain granular and fine cluster annotations derived from Harmony^38^ batch-corrected RNA and TotalVI-corrected ADT counts on the titrated dataset. scTriangulate was performed on the titration CITE-seq dataset using both SoupX-corrected RNA counts and TotalVI-corrected ADT normalized values and annotations from WNN and the cellHarmony titration dataset annotations. This analysis produced 89 integrated clusters. Refined final cell annotations for the titrated CITE-seq dataset were obtained using the same cellHarmony remapping protocol. UMAPs were produced in AltAnalyze using the default UMAP function, considering the top 60 cluster-specific marker genes as features rather than principal components. SCCAF stability scores were derived using the scTriangulate SCCAF function. Marker heat maps were obtained using the software MarkerFinder using either single cells or combined donor pseudobulks for each scTriangulate cell population. For differential ADT analyses, we applied an empirical Bayes moderated *t*-test (FDR corrected), as this robust procedure is typical for molecular ‘omics comparison analyses.

The AML CITE-seq samples using the 132-antibody cocktail were processed using the same protocol as the titrated CITE-seq controls (CellRanger, SoupX and TotalVI) and mapped to the titrated scTriangulate clusters using cellHarmony (default options, centroid alignment, correlationCutoff = 0). Differential ADT abundance analyses were performed in cellHarmony using the default testing procedure for each matched cell population for all cells in the p-LSC versus p-LSC + m-LSC samples comparison (empirical Bayes *t*-test *P* < 0.05, FDR corrected). Bone marrow scRNA-seq CPTT scaled count matrices were obtained from three prior described cohorts (*n* = 38 donors) from the author-provided count matrices and were also aligned to this titrated scTriangulate cluster with cellHarmony. cellHarmony-annotated cells from these 38 healthy bone marrow samples were projected into the reference UMAP coordinate space using the AltAnalyze ‘approximateUMAP’ function ^3,20,32^.

### ADT marker nomination and concentration selection

To prioritize oligonucleotide-conjugated CITE-seq antibodies in their analytical value scTriangulate transcriptionally defined cell populations, we trained an XGBoost classification model using ADT expression levels to predict scTriangulate clusters for each concentration tested in the titration. The gain in feature importance metric was then used to rank each antibody’s contribution to the prediction. Meanwhile, we checked the specificity of ADTs at a given titration concentration by UMAP and heat map visualization compared to isotype controls. Specifically, we considered an ADT underperforming if its signal was sparse within clusters that it was supposed to label (by XGBoost or based on the literature) or nonspecific if it exhibited an indistinguishable staining pattern as most isotype controls at the lowest concentration. Sixty-seven ADTs that ranked in the bottom 50% among all concentrations were examined to rescue well-established markers. Unknown markers within the consistent bottom 50% in XGboost and/or that were identified as nonspecific were excluded. For ADTs that exhibited dose-dependent signals, we chose the concentration that retained over 75% of the observed dynamic range after confirming that the increased concentration did not lead to nonspecific staining in irrelevant clusters. For example, a 2× concentration was selected for CD38 because it displayed a robust dose-dependent dynamic range across all donors (Fig. 2b), high expression in plasma cells and expression at distinguishable intermediate levels in CLPs but not HSCs (Fig. 2c). For ADTs that did not demonstrate a dose-dependent dynamic range, we concluded that all five concentrations used were either saturating or considerably below the optimal concentration. For those in the first scenario (saturating), we lowered the concentration for ADTs enriched in specific populations but exhibiting high levels of background signal as assessed by comparing to isotype controls; otherwise, we chose the 1× concentration. For example, CD29 stained nonspecifically at higher concentrations; therefore, we chose a 0.5× concentration (Extended Data Fig. 4c). In cases where an ADT appeared specific at select concentrations but showed few reads per cell and overall weak staining as indicated by its dynamic range, we consulted BioLegend for the concentration relative to the TotalSeq-A Human Universal Cocktail V1.0 and adjusted the concentration accordingly (for example, CD325, CD27, CD162, CD11c, CD55 and CD44, Extended Data Fig. 4a,b). Specific markers associated with more mature subsets not resolved in the progenitor atlas were excluded from the final titration, including several lymphocyte markers, ADTs targeting T cell receptors and immunoglobulins, as markers for lymphocyte clonality are not required to assess progenitor cell and broad lineage identity. BioLegend formulated and lyophilized 125 of 132 ADTs at specific concentrations (Supplementary Table 6) as a custom panel to enable subsequent validation.

### PE antibody titration

Human bone marrow cells were processed and magnetically enriched for CD34^+^ cells. Forty million CD34^–^ cells were mixed with three million CD34^+^ cells to better represent progenitor and mature lineage populations. Fifty thousand cells were stained for each concentration of 1:25, 1:50, 1:100, 1:200 and 1:400 (vol/vol antibody:staining buffer). Cells were settled on 96-well Laminar Wash plates (96-DC-CL-05, Curiox Biosystems) at 4 °C for 30 min and were washed on a Laminar Wash HT2000 System (Curiox Biosystems) at a setting of 15 cycles, wash rate of 10 μl s^–1^ and initial volume of 55 μl. Data were analyzed using an Automated Sample Loader on a five-laser Cytek Aurora full-spectrum flow cytometer (Cytek Biosciences) by adding a 96-well grid adaptor (Curiox Biosystems) onto the laminar wash plate. In total, 10,000 to 20,000 live cells were recorded, and FCS files were exported for analysis using FlowJo v10.8.1 software with the StainIndex v1.8.1 plugin (BD Biosciences). The optimal concentration for each PE antibody was selected by determining the maximum stain index. Stain indexes and final concentration information can be found in Supplementary Tables 13 and 14. Of note, the PE antibodies that overlapped with the backbone were titrated but not used in the Infinity Flow assay.

### Infinity Flow data generation

In total, 15 million bone marrow cells from each donor were stained for 30 min at 4 °C with a panel modified from the Cytek 20-Color AML Panel. The antibodies in the backbone were used at concentrations recommended by the manufacturer. After washing, 50,000 cells were aliquoted into each well of 96-well Laminar Wash plates (96-DC-CL-05, Curiox Biosystems) and stained with each titrated PE antibody for 30 min at 4 °C. Cells were then washed on a Laminar Wash HT2000 System (Curiox Biosystems) with a flow rate of 5 μl s^–1^, which reduced any physical stress-like drawbacks by exponential dilution of medium based on laminar flow rates. Direct well-to-SIT analysis on a five-laser Cytek Aurora through a Laminar Wash Direct Reading Grid (DC-GR02-96-M, Curiox Biosystems) allows recording upwards of 10,000–20,000 live cells with a 10-s mix time. Multiplate runs were possible through the application of Laminar Wash plates on a Cytek Aurora Plate Loader. The same set of FSP bead-based single-color controls were used to standardize the controls across all donors. FCS files were exported for analysis using FlowJo v10.8.1 software (BD Biosciences).

### Infinity Flow object generation and analysis

Live and single cells were gated from the unmixed FCS files (Extended Data Fig. 9a) and exported as inputs to pyInfinityFlow version 1.0.5. All parameters were included for analysis except for live/dead. Infinity marker expression was imputed using the default settings with the following modifications: --ratio_for_validation 0.5 and --n_events_combine 0. Infinity objects were imported into the FlowJo v10.8.1 software (BD Life Sciences) for analysis. All cell surface marker parameters were analyzed on a biexponential scale. Cells were gated for granulocytes, lymphocytes, blast cells, monocytes and stromal cells (CD45^–^) based on side scatter area and expression of CD45.

### Batch effect correction by cyCombine

Infinity Flow FCS objects were integrated across samples and with the CITE-seq ADT profiles using the cyCombine workflow. ADT values were normalized by centered log ratio (CLR) transformation. Batch effect correction was performed with the following settings: seed = 840, xdim = 8, ydim = 8, norm_method = rank and ties.method = average; the cofactor for spectral flow data was set to 6,000. Modifications to plot functions were made to output one plot per page. Modified code is supplied in our GitHub repository. The Earth Mover’s Distance plots were generated using the evaluate_emd function in CyCombine to assess variability among all nine separate donors before and after correction and for the pairwise comparison of each CITE-seq and Infinity Flow-assayed donor sample BMNC.

### Antigen detection consistency assessment

We integrated the cyCombine batch-corrected antibody profiles from CITE-seq and Infinity Flow for the four common donors profiled. To evaluate surface marker consistency across donors and technologies, we applied both expert curation of the obtained antibody distribution ridge plot and a prior described statistical comparison approach (*k*-sample Anderson–Darling test)^39^. Expert curation defined 79 surface markers consistent between CITE-seq and Infinity Flow and 40 surface markers consistent between all nine Infinity Flow profiled donors. In total, 35 markers were consistent based on ridge plot inspection across technology and donor (Supplementary Table 16). As an alternative approach, we applied the *k*-sample Anderson–Darling test implemented in the R package kSamples. Comparison of distributions of corrected flow data across the nine donors was performed by first randomly subsampling data from each donor to 2,500 observations. The function ad.test (method = ‘asymptotic’) was then used to compute the Anderson–Darling *t* value for each marker. Distributions of corrected flow and CITE-seq data within the four donors was performed by first randomly subsampling data from each donor/technology combination to 2,450 observations (approximately the minimum number of observations across the eight combinations). The function ad.test.combined (method = ‘asymptotic’) was then used to compute the Anderson–Darling *t* value (technology within donor) for each marker (Supplementary Table 17). This analysis assigns a score for each surface marker based on its relative consistency.

### Cell sorting for TSPAN33^+^C5L2^+^ cells and bulk culture c.f.u. assays

Total nucleated cells were isolated from unprocessed bone marrow product by layering 1:1 PBS:cell suspension over 15-ml Ficoll Paque Plus in SepMate-50 tubes, according to the manufacturer’s instructions. RBCs were lysed with PharmLyse (555899, BD). Cells were then labeled according to Supplementary Table 15 and sorted by a BD FACSAria II directly into culture medium consisting of MegaCult-C Medium Plus Lipids (04850, STEMCELL Technologies) supplemented with 3.0 U ml^–1^ recombinant human (rh) erythropoietin (rhEPO), 10 ng ml^–1^ rh interleukin-3 (rhIL-3), 10 ng ml^–1^ rhIL-6, 25 ng ml^–1^ rh stem cell factor (rhSCF), 50 ng ml^–1^ rh thrombopoietin (rhTPO), 20 ng ml^–1^ rh granulocyte colony-stimulating factor (rhG-CSF), 20 ng ml^–1^ rh macrophage colony-stimulating factor (M-CSF) and 20 ng ml^–1^ rhGM-CSF. The sorted cells were then mixed with collagen solution (04902, STEMCELL Technologies) to a final concentration of 1.2 mg ml^–1^, plated in six-well plates and incubated at 37 °C with 5% CO_2_ for 1 week. Colonies were stained in situ 6 days after plating using antibodies according to Supplementary Table 15. Colony assays were imaged the next day using an ImageExpress-4 (Molecular Devices) to produce high-resolution scans of each well, which were then processed in ImageJ for subsequent colony scoring.

### Cell sorting for MEPs and bulk culture c.f.u. assays

Primary human CD34^+^ cells, obtained from the Yale Cooperative Center of Excellence in Hematology, were stained with antibodies according to Supplementary Table 15. Human MEP (DAPI^–^Lin^–^CD34^+^CD41a^–^CD45RA^−^CD135^–^CD110^–^CD38^mid^)^40^, MEP CD133^low^, MEP CD133^mid^ and MEP CD133^hi^ cells were sorted on a BD FACSAria. The c.f.u. analysis was performed as previously described^41^. Briefly, MEPs, MEP CD133^low^, MEP CD133^mid^ and MEP CD133^hi^ cells were cultured in MegaCult-C Medium Plus Lipids (04850, STEMCELL Technologies) mixed with collagen solution (04902, STEMCELL Technologies) to a final concentration of 1.2 mg ml^–1^ with 3.0 U ml^–1^ rhEPO, 10 ng ml^–1^ rhIL-3, 10 ng ml^–1^ rhIL-6, 25 ng ml^–1^ rhSCF, 50 ng ml^–1^ rhTPO, 20 ng ml^–1^ rhG-CSF, 20 ng ml^–1^ rhM-CSF and 20 ng ml^–1^ rhGM-CSF at 37 °C with 5% CO_2_. At day 6 after plating, colonies were stained in situ with c.f.u. staining panel antibodies in Supplementary Table 15 diluted in 300 μl of PBS (1:100 dilution) per well of a six-well plate. At day 7, six-well plates were imaged using a Molecular Devices ImageXpress Micro 4 microscope to produce high-resolution whole-well scans at ×40 magnification. All images were then processed using ImageJ, and all colonies were counted manually from processed images.

### MEP indexed sorting and c.f.u. assays

Primary human CD34^+^ cells were obtained from the Yale Cooperative Center of Excellence in Hematology. Cells were stained with antibodies according to Supplementary Table 15 and index sorted on a BD FACSAria II as singlet, live, CD34^+^CD45RA^–^CD135^–^ events using single-cell purity mode into 384-well plates (3765, Corning) that were prefilled with 80 μl per well of culture medium. Culture medium was composed of IMDM (21056023, Gibco) supplemented with 0.1 mM β-mercaptoethanol, 20% BIT 9500 (09500, STEMCELL Technologies), 40 μg ml^–1^ human low-density lipoprotein (02698, STEMCELL Technologies), 1× GlutaMAX (35050061, Gibco), human SCF (25 ng ml^–1^), human IL-3 (10 ng ml^–1^), human IL-6 (10 ng ml^–1^), human GM-CSF (20 ng ml^–1^), human M-CSF (20 ng ml^–1^), human G-CSF (20 ng ml^–1^), human TPO (50 ng ml^–1^; T1003.1, ConnStem) and human EPO (3 U ml^–1^; Amgen). Cells were incubated for 14 days at 37 °C with 5% CO_2_. To fluorescently label and identify lineages produced in colonies, antibodies were diluted into PBS according to Supplementary Table 15, added to each well on day 13 of culture and incubated overnight to bind. Plates were imaged with phase contrast and fluorescence using an ImageXPress Micro 4 microscope and sampled for flow cytometric analysis using a four-laser BD Fortessa (BD) equipped with a high-throughput sampler. Images were processed using FIJI. All colonies were scored manually by analyzing fluorescence channels and comparing their flow cytometry profiles. Flow cytometry data were processed using FlowJo v10.9.0.

### CD235a and CD326 erythroid sort

Human bone marrow was stained with a lineage cocktail using FITC-conjugated antibodies to CD2, CD7, CD11b and CD14 and erythroid markers CD71-PE (334105, BioLegend), CD326-APC (324207, BioLegend) and CD235a-BV421 (349131, BioLegend) and DAPI to identify viable cells. Cell sorting was performed on a FACSAria II to collect four populations (CD71^+^CD326^+^CD235a^–^, CD71^+^CD326^+^CD235a^+^, CD71^+^CD326^–^CD235a^+^ and CD71^–^CD326^–^CD235a^+^) or sorted on an MA900 where cells were stained with Percp/Cy5.5-conjugated antibodies to CD2, CD7, CD11b and CD14 and erythroid markers CD71-FITC (334103, BioLegend), CD326-APC (BioLegend, clone 9C4) and CD235a-BV421 (BioLegend, clone HI264).

### Cytospin staining

Cytospin slides were prepared from each cell population with a Thermo Scientific Cytospin 4 set to spin at 500 rpm for 5 min onto Superfrost Plus microscope slides and stained with Differential Quik III (Polysciences) or set to spin at 900 rpm for 3 min onto VWR HistoBond slides (16004-406, VWR) and stained with Camco Stain Pak (702, Cambridge Diagnostic Products). Cellular morphology and characterization of each population were assessed with an upright light microscope with a ×40 objective.

### RShiny app development and analysis

The multimodal Azimuth bone marrow reference RShiny interface was built following the Azimuth v0.4.6 instructions (https://github.com/satijalab/azimuth) using the neighbors from the titrated RNA data restricted to the top MarkerFinder marker genes (https://github.com/nsalomonis/Human-Bone-Marrow-Titration-Atlas/tree/main/build_azimuth). CITE-seq RNA counts^34^ were scaled and normalized as CPTT with clusters defined for three different annotation levels based on multimodal scTriangulate clusters. The Azimuth browser was parameterized to impute ADT concentrations per cell as an optional input for user analysis through the hosted Azimuth web interface. The AML CITE-seq h5ad read counts file was analyzed using the local implementation of the Azimuth R object following import. Per-sample cell frequency differences in p-LSC- and m-LSC-only biopsies were determined by a Student’s *t*-test *P* value (*P* ≤ 0.05). A ShinyCell viewer for the healthy bone marrow compendium was generated using a formatted h5ad counts matrix with corresponding sample/cell-level metadata for both the optimized titrated and titration datasets.

### Reporting summary

Further information on research design is available in the Nature Portfolio Reporting Summary linked to this article.

## Online content

Any methods, additional references, Nature Portfolio reporting summaries, source data, extended data, supplementary information, acknowledgements, peer review information; details of author contributions and competing interests; and statements of data and code availability are available at 10.1038/s41590-024-01782-4.

### Supplementary information

Reporting Summary

Supplementary Table Supplementary Tables 1–19.

### Source data

Source Data Fig. 3 Data for correlation plot (**h**) and heat maps (**i** and **j**).

Source Data Fig. 4 Actual sorting gates and c.f.u. counts for **e** and **i** and cell counts of **f**.

Source Data Fig. 5 Values in **j** heat map.

Source Data Fig. 6 T-test examination of cell composition between p-LSC and m-LSC AML samples.

Source Data Extended Data Fig. 7 Values in a heat map.

Source Data Extended Data Fig. 9 Actual sorting gates and c.f.u. counts/morphology counting of **d**, **f** and **i** and cell counts of **j**.

## Data Availability

Raw sequencing FASTQ files and the processed HDF5 (h5) matrix have been deposited in the Gene Expression Omnibus database under accession code GSE245108. CITE-seq data generated by Pei et al. were accessed via the accession ID GSE232559. All imputed Infinity Flow objects are available in the FlowRepository under ID FR-FCM-Z6UQ. An interactive web portal with the associated Azimuth instance and data visualization tools is available at https://altanalyze.org/MarrowAtlas/. Interactive data visualization and analysis tools for the CITE-seq bone marrow single-cell datasets are provided at https://altanalyze.org/MarrowAtlas/. Source data are provided with this paper.

## Code availability

The processed HDF5 (h5) matrix and code have been deposited in Synapse under project SynID syn52600803. Source code and analysis scripts are available at GitHub (https://github.com/nsalomonis/Human-Bone-Marrow-Titration-Atlas).
