# Supplementary material for: An immunophenotype-coupled transcriptomic atlas of human hematopoietic progenitors
Source: Nat Immunol. 2024 Mar 21;25(4):703–15. doi: 10.1038/s41590-024-01782-4 (PMC11003869; doi:10.1038/s41590-024-01782-4)
Supplement: Supplementary file 1 — Reporting Summary [file 41590_2024_1782_MOESM1_ESM.pdf]

nature portfolio

Corresponding author(s): NI-RS36778

Last updated by author(s): Nov 8, 2023

# Reporting Summary

Nature Portfolio wishes to improve the reproducibility of the work that we publish. This form provides structure for consistency and transparency in reporting. For further information on Nature Portfolio policies, see our Editorial Policies and the Editorial Policy Checklist.

## Statistics

For all statistical analyses, confirm that the following items are present in the figure legend, table legend, main text, or Methods section.

| n/a | Confirmed | |
|---|---|---|
| ☐ | ☒ | The exact sample size (*n*) for each experimental group/condition, given as a discrete number and unit of measurement |
| ☐ | ☒ | A statement on whether measurements were taken from distinct samples or whether the same sample was measured repeatedly |
| ☐ | ☒ | The statistical test(s) used AND whether they are one- or two-sided<br>*Only common tests should be described solely by name; describe more complex techniques in the Methods section.* |
| ☐ | ☒ | A description of all covariates tested |
| ☐ | ☒ | A description of any assumptions or corrections, such as tests of normality and adjustment for multiple comparisons |
| ☐ | ☒ | A full description of the statistical parameters including central tendency (e.g. means) or other basic estimates (e.g. regression coefficient) AND variation (e.g. standard deviation) or associated estimates of uncertainty (e.g. confidence intervals) |
| ☐ | ☒ | For null hypothesis testing, the test statistic (e.g. *F*, *t*, *r*) with confidence intervals, effect sizes, degrees of freedom and *P* value noted<br>*Give P values as exact values whenever suitable.* |
| ☒ | ☐ | For Bayesian analysis, information on the choice of priors and Markov chain Monte Carlo settings |
| ☐ | ☒ | For hierarchical and complex designs, identification of the appropriate level for tests and full reporting of outcomes |
| ☒ | ☐ | Estimates of effect sizes (e.g. Cohen's *d*, Pearson's *r*), indicating how they were calculated |

*Our web collection on statistics for biologists contains articles on many of the points above.*

## Software and code

Policy information about availability of computer code

| | |
|---|---|
| Data collection | Sony Cell Sorter Software (3.1.1), BD FACSDiva Software (V9.0.1), 10X Genmoics Chromium X (1.2.1), SpectralFlo(3.0.3), MetaXpress 6 (6.3.55) |
| Data analysis | cellranger(6.1.2), FlowJo v10.8.1 and V 10.9.0, pyInfinityFlow (v1.0.5), SoupX(1.6.0), cyCombine (0.2.15), XGboost (1.7.6), scTriangulate (0.13.0), scvi-tools (0.16.0), AltAnalyze (2.1.4), Seurat (V4), NIS-Elements Viewer(4.2), imageJ (1.54g), customized code are in GitHub repository. |

For manuscripts utilizing custom algorithms or software that are central to the research but not yet described in published literature, software must be made available to editors and reviewers. We strongly encourage code deposition in a community repository (e.g. GitHub). See the Nature Portfolio guidelines for submitting code & software for further information.

## Data

Policy information about availability of data

All manuscripts must include a data availability statement. This statement should provide the following information, where applicable:

- Accession codes, unique identifiers, or web links for publicly available datasets
- A description of any restrictions on data availability
- For clinical datasets or third party data, please ensure that the statement adheres to our policy

GEO: Raw sequencing FASTQ files and processed HDF5 (h5) matrix are available on Gene Expression Omnibus database (accession number GSE245108, https://www.ncbi.nlm.nih.gov/geo/query/acc.cgi?acc=GSE245108), in compliance with donor consents.

Synapse: processed HDF5 (h5) matrix and codes are available under Project SynID: syn52600803 (https://www.synapse.org/#!Synapse:syn52600803/wiki/625668)
FlowRepository: All imputed Infinity Flow objects are available under ID: FR-FCM-Z6UQ (http://flowrepository.org/experiments/7130).
GitHub: Source code and analysis scripts are available in GitHub (https://github.com/nsalomonis/Human-Bone-Marrow-Titration-Atlas).
Azimuth: An interactive web portal with the associated Azimuth instance and data visualization tools are available at https://altanalyze.org/MarrowAtlas/.
ShinyCell: An interactive data visualization and analysis tools for the CITE-Seq bone marrow single-cell datasets is provided at https://altanalyze.org/MarrowAtlas/.

## Human research participants

Policy information about studies involving human research participants and Sex and Gender in Research.

| | |
|---|---|
| Reporting on sex and gender | We use the term "sex" consistently throughout the manuscript. All sex were self reported and collected by Lonza with donors' consent. All donor information is listed in supplementary table 1 or source data files. |
| Population characteristics | We characterize donors by age, sex and race. All of our donors are healthy, young(<35) and non-smoker at the time of collection. Donor information was collected by Lonza with donors' consent. |
| Recruitment | The bone marrow collection process is a three-step process:<br>1. donor screening<br>2. bone marrow collection<br>3. day after donation survey<br>for details please see consent forum |
| Ethics oversight | The program protocol, including the consent form and questionnaire, are reviewed and have been approved by Salus IRB (00074), an Association for the Accreditation of Human Research Protection Programs accredited Commercial Institutional Review Board. |

Note that full information on the approval of the study protocol must also be provided in the manuscript.

# Field-specific reporting

Please select the one below that is the best fit for your research. If you are not sure, read the appropriate sections before making your selection.

☒ Life sciences ☐ Behavioural & social sciences ☐ Ecological, evolutionary & environmental sciences

For a reference copy of the document with all sections, see nature.com/documents/nr-reporting-summary-flat.pdf

# Life sciences study design

All studies must disclose on these points even when the disclosure is negative.

| | |
|---|---|
| Sample size | CITE-seq data was collected on samples from 4 females and 4 males. Infinity Flow data collected on samples from 5 females and 4 males. In vitro validation data collected from 1 male and 2 females. Sex was self-reported |
| Data exclusions | No samples was excluded. Low quality cells were removed by QC metrics (see Methods) |
| Replication | Three replicates from at least two donors in vitro validation showed consistent results between study sites (Yale and CCHMC). |
| Randomization | We intentionally balance our cohort in each experiment for both sex and race (black and white) |
| Blinding | In vitro validation experiments were blinded as the labeling for slides and tubes were masked when used for counting |

# Reporting for specific materials, systems and methods

We require information from authors about some types of materials, experimental systems and methods used in many studies. Here, indicate whether each material, system or method listed is relevant to your study. If you are not sure if a list item applies to your research, read the appropriate section before selecting a response.

## Materials & experimental systems

| n/a | Involved in the study |
|---|---|
| ☐ | ☒ Antibodies |
| ☒ | ☐ Eukaryotic cell lines |
| ☒ | ☐ Palaeontology and archaeology |
| ☒ | ☐ Animals and other organisms |
| ☒ | ☐ Clinical data |
| ☒ | ☐ Dual use research of concern |

## Methods

| n/a | Involved in the study |
|---|---|
| ☒ | ☐ ChIP-seq |
| ☐ | ☒ Flow cytometry |
| ☒ | ☐ MRI-based neuroimaging |

# Antibodies

Antibodies used

Antibodies catalog number, concentration/dilution used, lot number, RRID and titration results are in supplementary tables.

Validation

RRID for antibodies are provided in supplementary tables. Antibody species reactivity is validated typically using flow cytometry using purified antibodies on biologically relevant samples from target species. All antibodies used in this experiment with the exception of isotype controls have human reactivity. Validation of TotalSeq™ products is a separate process from quality testing (QC) of subsequently manufactured lots. Generally, clone-specificity of TotalSeq™ antibodies is tested using flow cytometry on primary origin samples of biological relevance from multiple donors and orthogonally compared with pure and fluorescent conjugate validation data to establish acceptance specifications. Quality testing of subsequent lot is conducted in reference to the original validation data according to specifications. Additionally, catalog TotalSeq™ antibody cocktail products are both validated quality tested using the relevant single-cell Multiomics platform using various samples of biological relevance of primary origin from multiple donors.

# Flow Cytometry

## Plots

Confirm that:

☒ The axis labels state the marker and fluorochrome used (e.g. CD4-FITC).

☒ The axis scales are clearly visible. Include numbers along axes only for bottom left plot of group (a 'group' is an analysis of identical markers).

☒ All plots are contour plots with outliers or pseudocolor plots.

☒ A numerical value for number of cells or percentage (with statistics) is provided.

## Methodology

Sample preparation

Samples are bone marrow total nucleated cells isolated (ficol) from overnight shipped fresh human bone marrow. Staining conditions and antibody used are in method

Instrument

Cytek® Aurora (5 Laser 16UV-16V-14B-10YG-8R), Sony MA900, BD FACSAria™ Fusion Flow Cytometer (5 laser)

Software

SpectralFlo(3.0.3), Sony Cell Sorter Software (3.1.1), BD FACSDiva Software (V9.0.1),

Cell population abundance

Cells frequency of each sorted population were shown in representative flow gates. Due to the low number of cells sorted and study design (directly sorting into culture mix), we were not able determine post-sort fraction cells purity eg. staining with antibody targeting other epitopes of the same antigen.

Gating strategy

gating based on FMO or arbitrary percentage (high, mid, low) for sorting C5L2/TSPAN33 cells

☒ Tick this box to confirm that a figure exemplifying the gating strategy is provided in the Supplementary Information.
